# Epigenome alterations in aortic valve stenosis and its related left ventricular hypertrophy

**DOI:** 10.1186/s13148-017-0406-7

**Published:** 2017-10-03

**Authors:** Igor Gošev, Martina Zeljko, Željko Đurić, Ivana Nikolić, Milorad Gošev, Sanja Ivčević, Dino Bešić, Zoran Legčević, Frane Paić

**Affiliations:** 1 Department of Surgery, University of Rochester Medical center, Rochester, NY USA; 2 Department of Cardiology, Clinical Unit of Internal Medicine, Clinical Hospital Merkur, Zajćeva 19, 10 000 Zagreb, Croatia; 3 Department of Cardiac Surgery, University Hospital Center Zagreb, Kišpatićeva 12, 10 000 Zagreb, Croatia; 4 Division of Cardiovascular Medicine, Brigham and Women’s Hospital, 75 Francis Street, Boston, MA 02115 USA; 5 School of Medicine, University of Josip Juraj Strossmayer, Trg Svetog trojstva 3, 31 000 Osijek, Croatia; 6 Department of Physiology, School of Medicine, University of Zagreb, Šalata 3, 10 000 Zagreb, Croatia; 7 Laboratory for Epigenetics and Molecular Medicine, Department of Biology, School of Medicine, University of Zagreb, Šalata 3, 10 000 Zagreb, Croatia

**Keywords:** Aortic stenosis, DNA methylation, Histone modification, Chromatin remodeling, lncRNA, miRNA, Epigenome, Epigenetics

## Abstract

Aortic valve stenosis is the most common cardiac valve disease, and with current trends in the population demographics, its prevalence is likely to rise, thus posing a major health and economic burden facing the worldwide societies. Over the past decade, it has become more than clear that our traditional genetic views do not sufficiently explain the well-known link between AS, proatherogenic risk factors, flow-induced mechanical forces, and disease-prone environmental influences. Recent breakthroughs in the field of epigenetics offer us a new perspective on gene regulation, which has broadened our perspective on etiology of aortic stenosis and other aortic valve diseases. Since all known epigenetic marks are potentially reversible this perspective is especially exciting given the potential for development of successful and non-invasive therapeutic intervention and reprogramming of cells at the epigenetic level even in the early stages of disease progression. This review will examine the known relationships between four major epigenetic mechanisms: DNA methylation, posttranslational histone modification, ATP-dependent chromatin remodeling, and non-coding regulatory RNAs, and initiation and progression of AS. Numerous profiling and functional studies indicate that they could contribute to endothelial dysfunctions, disease-prone activation of monocyte-macrophage and circulatory osteoprogenitor cells and activation and osteogenic transdifferentiation of aortic valve interstitial cells, thus leading to valvular inflammation, fibrosis, and calcification, and to pressure overload-induced maladaptive myocardial remodeling and left ventricular hypertrophy. This is especcialy the case for small non-coding microRNAs but was also, although in a smaller scale, convincingly demonstrated for other members of cellular epigenome landscape. Equally important, and clinically most relevant, the reported data indicate that epigenetic marks, particularly certain microRNA signatures, could represent useful non-invasive biomarkers that reflect the disease progression and patients prognosis for recovery after the valve replacement surgery.

## Background

Aortic valve stenosis (AS) is the most frequent heart valve disease among adults in the Western societies with ever increasing prevalence due to the rapidly aging population [1–3]. According to the recent population-based studies performed in Europe and North America the pooled prevalence of total and symptomatic severe AS cases in the general elderly population (≥ 75 years of age) is estimated to 12.4, and 3.4%, respectively [4]. Moreover, with current trends in the population demographics by the year 2050 there will be an estimated 2.1 million European and 1.4 million North American patients with symptomatic severe AS [4]. Furthermore, the even more pronounced demographic changes in Africa, Asia, and South America will further increase the absolute number of AS patients [2, 4, 5]. Therefore, in a very recent future, AS is likely to become a major health and economic burden facing the worldwide societies [2, 4, 5].

The major cause of AS is thickening, fibrosis, and calcification of a previously normal tricuspid valve (TAV) or a congenital bicuspid valve (BAV), while a historically prevailing rheumatic heart valve disease, and still the most common cause of AS in developing countries accounts for only 10% of diagnosed cases [2, 6, 7].

No matter the cause, development of AS starts with the risk of leaflet changes and progresses over many years from early lesions to subsequent narrowing (stenosis) of the aortic valve orifice [8]. During that time, the genetic predisposition or otherwise induced faulty valve repair system in concordance with continuous blood born mechanical forces and proatherogenic risk factors (i.e., hyperhomocysteinemia, hyperlipidemia, abnormal calcium metabolism, smoking, metabolic syndrome, diabetes, hypertension, chronic renal failure, male gender, age) leads to endothelial dysfunctions followed by disruption of the subendothelial basement membrane, extracellular accumulation of plasma-derived atherogenic lipoproteins and infiltration/activation of monocyte-macrophage cells, mast cells, and T lymphocytes [3, 9–14]. That leads to intracellular lipid deposition, generation of oxidative stress with accumulation of oxidized lipids and apolipoproteins, foamy cells formation, and upregulation of various pro-fibrotic and pro-inflammatory factors with concomitant inhibition of plasma derived or locally presents anti-calcific proteins. Acting together, these factors promote extensive extracellular matrix remodeling, and activation of signaling pathways that promote neovascularization, inflammation and calcification [3, 9–14]. Concomitant transformation of normally quiescent valvular interstitial cells (qVICs) to active myofibroblastic (aVICs) phenotype in the valve interstitium and their subsequent differentiation to osteoblast-like cells (obVICs) with activation of pro-osteogenic signaling pathways is thought to be one of the central mechanisms contributing to the initiation and progression of AS [15]. In addition, a subset of aortic valvular endothelial cells (VECs) undergoing endothelial- to-mesenchymal transition (EMT) and/or circulating osteoprogenitor cells (COPCs) may also contribute to valvular calcification/ossification either by redifferentiating to an osteoblast-like phenotype or by promoting VICs activation through paracrine signaling [16–18].

Based on this timely dependent change of tissue organization, the disease has been divided in two successive functional categories: aortic valve sclerosis (ASc) and aortic valve stenosis. ASc represents the initial, clinically mostly silent stage of disease with calcification and mild fibrous thickening of the aortic leaflets without marked functional obstruction of left ventricular outflow [8, 19]. Contrary, aortic valve stenosis as the more advanced stage of the disease comprise serious impairment of leaflet motion with subsequent obstruction of blood flow resulting in maladaptive left ventricular hypertrophy (LVH), myocardial fibrosis (MF), and a propensity for systolic and diastolic dysfunction and heart failure (HF) [3, 8, 20].

Currently there are no effective pharmacological remedies to prevent or slow the progression of AS and aortic valve replacement (AVR) either surgical or less invasive transcatheter (TAVR) approach is still the only clinical therapy at hand for its successful treatment [21, 22, 23]. Thus, a better understanding of mechanisms involved in the pathogenesis and progression of AS could lead to novel diagnostic, prognostic and therapeutic targets and eventual development of noninvasive therapeutic options.

Recent observations suggest that the full pathological spectrum of AS lesions cannot be entirely accounted for by hereditary predisposition or growing list of differentially expressed genes. Moreover, traditional genetic views do not sufficiently explain the well-known link between AS, proatherogenic risk factors, and disease-prone environmental influences. Thus, it has become clear that other regulatory mechanisms are essential, and a compelling argument for an epigenetic contribution is rapidly emerging [11, 14].

Epigenetics refers to mitotically and meiotically stable (heritable), and functionally relevant DNA and chromatin modifications that are not caused by alterations (mutations) in the primary DNA sequence itself [24]. They can be either inherited or accumulated throughout a lifetime. Furthermore, given that each cell in our organism contains the identical genomic DNA the epigenetic mechanisms are fundamental to proper lineage commitment, cell fate determination, organogenesis, and ultimately, whole body homeostasis. More specifically, by altering the chromatin architecture and the accessibility of DNA coding and regulatory regions they orchestrate the spatiotemporal gene expression in a cell-type and even allele-specific (maternally or paternally imprinted loci) manner [25].

**Fig. 1:**
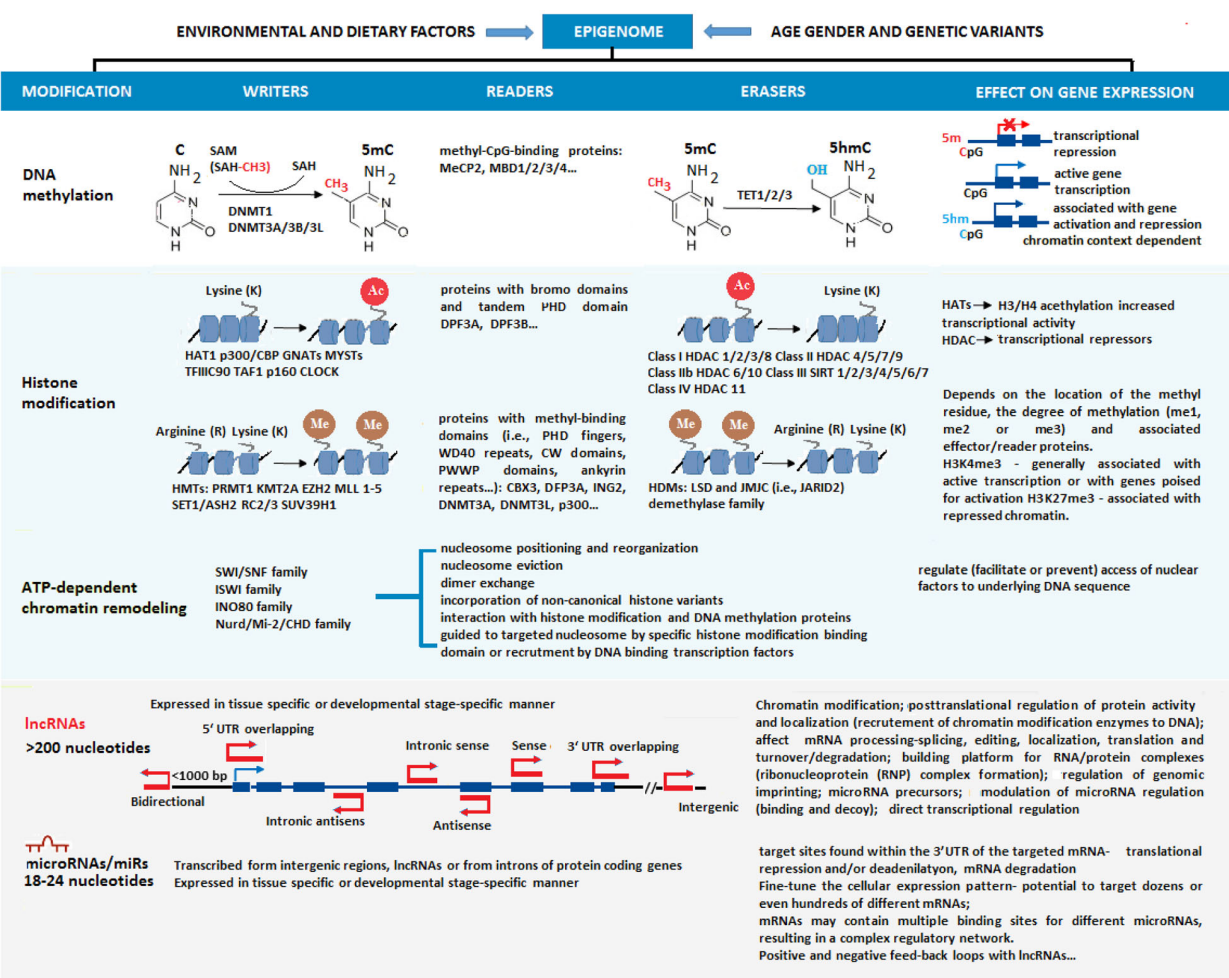
Major epigenetics mechanisams acting in mammalian cells. Presented are the four epigenetic mechanisams and their major impact on cellular gene regulation. Some writers (proteins that establish epigenetic marks) and riders (proteins that interpret epigenetic marks) are also illustrated. *DNMT* DNA methyl transferase, *CBX3* Chromobox 3, *CLOCK* clock circadian regulator, *DPF3A* double PHD fingers 3a, *5hm* 5 methyl cytosine, *HAT1* histone acetyltransferase 1, *HMT* histone methyl transferase*, ING2* inhibitor of growth family member 2*, KMT2A* lysine methyltransferase 2a, *MeCP2* methyl CpG binding protein 2, *MBD1* methyl-CpG-binding domain protein 1, *MLL* 1–5 family of lysine methyltransferases, *MYSTs* family of histone acetyltransferase, *p300* histone acetyltransferase P300, *PRMT1* protein arginine methyltransferase 1, *p160* MYB binding protein 1a, *SAM* S-adenosyl methionine, *SET1/ASH2* histone methyltransferase complex, *SUV39H1* histone-lysine N-methyltransferase

Four major epigenetic mechanisms (Fig. 1) have been characterized: DNA methylation and hydroxymethylation, covalent histone modifications and incorporation of histone variants, ATP-dependent chromatin remodeling, and chromatin and gene regulation by non-coding RNAs (ncRNAs) including small micro RNAs (microRNAs/miRs) and long non-coding RNAs (lncRNAs) [26–31]. These mutually interdependent epigenetic alterations, collectively named the *epigenome*, have profound effects on cellular repertoire of active genes [32, 33]. Furthermore, the well-known characteristics of epigenetic regulatory mechanisms: stability, adaptability, and reversibility, are all equally important in maintaining and changing cellular phenotype and function in both health and disease.

First, through epigenetic modifications genes are switched *on* and *off* in a more durable fashion than by any other mechanisms of gene regulation. Secondly, epigenetic alterations undergo dynamic changes during development and in response to the various nutritional, behavioral and environmental stimuli [34]. Notably, early changes of epigenetic regulatory mechanisms caused by fetal environment may influence the adult phenotype, including an individual’s susceptibility to cardiovascular diseases (CVD) later in life, and the late onset of CVDs may well be linked to age-related alterations of epigenetic marks [35–39]. Finally, and clinically most relevant, all known epigenetic marks are reversible, thus opening the possibility for prophylactic or therapeutic intervention and reprogramming of cells even in the early stages of disease progression.

Herein, we provide a comprehensive overview of currently known epigenetic mechanisms involved in the control of gene expression in the native and infiltratory aortic valve cells, and discusses their roles in the pathogenesis and the progression of AS. Myocardial epigenome alterations due to pressure overload (PO) LVH induced by AS will also be covered.

### DNA methylation

The data examining the role of DNA methylation changes in etiology of AS are only beginning to emerge.

For instance, Nwachukwu et al. reported dramatically increased levels of *DNMT3B* [DNA (Cytosine-5-)-methyltransferase 3 beta] expression in human AS compared to control valves, that was associated with an increase in global DNA methylation [40]. Furthermore, through site-specific methylation analysis, they identified more than 6000 differentially methylated sites between normal and stenotic valves [40]. Interestingly AS leaflets also showed four times higher expression of pro-osteogenic marker osterix (*SP7/OSX*) [40].

Furthermore, Sritharen et al. showed that genetic inactivation of *DNMT3B* protects against activation of osteogenic pathways and slows the progression of AS [41]. In their experiment, aortic valves from haploinsufficient mice (*LDLR*−/−/*APOB100/100*, *DNMT3B*+/−) showed increased expression of *FABP4* (fatty acid binding protein 4; opposes osteogenesis) and *SMAD6* (SMAD family member 6; opposes bone morphogenetic protein /BMP/ signaling) gene products while expression of the osteogenic genes *MSX2* (MSH homeobox 2) and *SPP1/OPN* (secreted phosphoprotein 1; bone sialoprotein I, osteopontin) were substantially reduced [41].

Additional proof for the involvement DNA methylation changes in etiology of AS was reported by Radhakrishna et al. [42]. Their comparative DNA methylation analysis of neonatal dried blood spots obtained from newborns with AS disease and gestational-age matched controls revealed 59 significantly altered (hypomethylated or dimethylated) CpG methylation sites in the coding and/or promoter regions of 52 genes [42]. More specifically, they observed a significant methylation changes in *APOA5* (apolipoprotein A5), *PCSK9* (proprotein convertase and subtilisin/knexin-type 9), *DUSP27* (dual-specificity phosphatase 27), *RUNX1* (runt-related transcription factor 1), and *TXNRD2* (thioredoxin reductase 2) gene thus concluding that their altered expression is likely responsible for congenital AS [42]. Importantly, many of these differentially methylated CpG sites demonstrated good to excellent diagnostic accuracy for the prediction of AS status, thus raising the possibility to be used as molecular screening markers for non-invasive risk estimation and disease detection [42].

Importantly, Gilsbach et al. reported that altered methylation pattern of CpG sites may also contribute to regulation of LVH as a response to chronic PO induced by AS [43]. They identified 1280 differentially methylated CpG sites in myocardial biopsies from AS patients with cardiac hypertrophy (CH) and 1365 CpG sites in patients with HF, with 523 of them significantly altered in both patient groups [43]. In addition, 496 of these differentially methylated CpG sites were concordantly altered both in hypertrophic and in HF tissue samples [43].

The first piece of evidence for the role of gene specific alteration of DNA methylation marks in AS was reported by *Nagy and Back* [44]. They showed that treatment of human AVICs with the DNA methyltransferase inhibitor 5-Aza-2′-deoxycytidine increases *ALOX5/5LO* (5-lipoxygenase) transcriptional levels and the production of the proinflammatory mediator LTB4 (leukotriene B4) [44]. These in vitro findings were confirmed in surgically explanted calcified aortic valves exhibiting reduced promoter methylation of *ALOX5* accompanied with significantly higher transcriptional levels compared with non-calcified valve tissue [44]. The same group has also previously reported that the local upregulation of 5-lipoxygenase pathway (*ALOX5, ALOXAP/FLAP/5-LO* activating protein, *LTA4H*/Leukotriene A4 hydrolase and *LTC4S*/Leukotriene C4 synthase) in human aortic valves leads to leukotriene-induced effects on aortic VICs (enhanced leukocyte recruitment, inflammation, increased reactive oxygen species /ROS/ production, LTB4-induced matrix metalloproteinase /MMP/ secretion, matrix remodeling, and calcification) and correlates significantly with the expression of osteogenic marker genes (*BMP2/6* and runt-related transcription factor/*RUNX2*) and severity of stenosis [45].

Gene-specific DNA-methylation changes in human AVICs were also reported in promoter region of *H19* (imprinted maternally expressed non-protein coding transcript) by Hadji et al. [46]. They showed that promoter region of this lncRNA is heavily methylated in healthy aortic valves resulting with no expression of its transcripts. Contrary, promoter hypomethylation observed in stenotic valves leads to increased *H19* expression which, in turn, decreases *NOTCH1* transcription (prevents the recruitment of P53 to *NOTCH1* promoter), and consequently increases the level of NOTCH1 downstream targets *RUNX2*, *BMP2*, and *BGLAP/OCN* (bone gamma-carboxyglutamate (Gla) protein; osteocalcin) thus promoting osteogenic reprograming of AVICs [46].

Finally, reduced expression of *EGFR* (epidermal growth factor receptor) associated with hypermethylation and dysregulation of its 5-hydroxymethylation pattern has also been linked with abnormal valve differentiation leading to the calcific AS and LVH in mice [47]. Since EGFR protein normally suppresses BMP pathway, attenuation of EGFR signaling may predispose differentiation of VICs to a calcifying cell phenotype [47].

Data accumulating from other tissues also suggest that DNA methylation may be involved in transcriptional regulation of genes implicated in AS, especially the ones involved in osteoblastic transdifferentiation of VICs.

For example, it is well known that obVICs are characterized by markers of osteoblastic differentiation, such as induction of *ALP* (alkaline phosphatase) [48]. Interestingly, DNA methylation plays important role in regulation of *ALP* expression in cells of the osteoblastic lineage, especially in its progressive transcriptional silencing during the osteoblasts to osteocyte transition [49, 50]. Notably, the degree of *ALP* promoter methylation is inversely associated with the transcriptional levels of ALP positive cells, with osteocytes, which do not express *ALP* showing high CpG island methylation [49].

Recent studies have also identified *SOST* (sclerostin; regulates osteoblast activity and serves as a Wnt /wingless-type MMTV integration site family member/pathway antagonist and a potent inhibitor of bone formation) gene expression as a novel marker of valvular calcification [51–53]. Notably, AS patients have significantly higher serum levels of *SOST* protein when compared to healthy subjects [51, 54]. Furthermore, tissues close to the calcified regions exhibit positive sclerostin staining, which is not observed in non-calcified control valves. In addition, *SOST* mRNA is significantly upregulated in calcified valves compared to non-calcified leaflets [51]. This increase in the *SOST* mRNA and protein levels is accompanied by the expression of prototypic markers (*RUNX2 and BGLAP/OCN*) of osteogenic transdifferentiation [51].

Importantly, SOST protein is produced by osteocytes and not osteoblasts and is regulated by DNA methylation during the final stage of osteoblast-to-osteocyte transition [55]. In addition, the *SOST* methylation marks are inversely correlated with its expression in osteoblastic cells but in this case with heavily methylated promoter in osteoblasts, which do not express SOST, while this same region is largely hypomethylated in human osteocytes [55–57].

Furthermore, the promoter regions of *DLX5* and *OSX* genes that exhibit increase expression in human VICs were also found methylated in non-osteogenic and unmethylated in osteogenic cell lines expressing these genes [58]. In addition, Arnsdorf et al. demonstrated that mechanical stimulation, essential for maintaining bone homeostasis, may reduce DNA methylation at the *SPP1/OPN* (osteopontin) promoter accompanied with corresponding increase in gene expression [59]. Interestingly, significant upregulation of *SPP1* expression was found in human AS valves in relation to preoperative transvalvular pressure gradient as well as in cultured VICs in relation to the mechanical strain applied [60, 61].

However, it should be emphasized that even though obVICs and cells of osteoblastic lineage share similar phenotypic markers, the applicability of the above findings to AS is currently unknown and remains largely to be established. A side by side comparison between VICs and several cell types in different stages of osteoblastic lineage commitment and differentiation performed by Monzack and Masters have shown stark differences in both the level and pattern of gene expression [62]. Whether they also include the noticeable dissimilarity in patterns of epigenetic gene regulation is currently quite unknown.

**Fig. 2:**
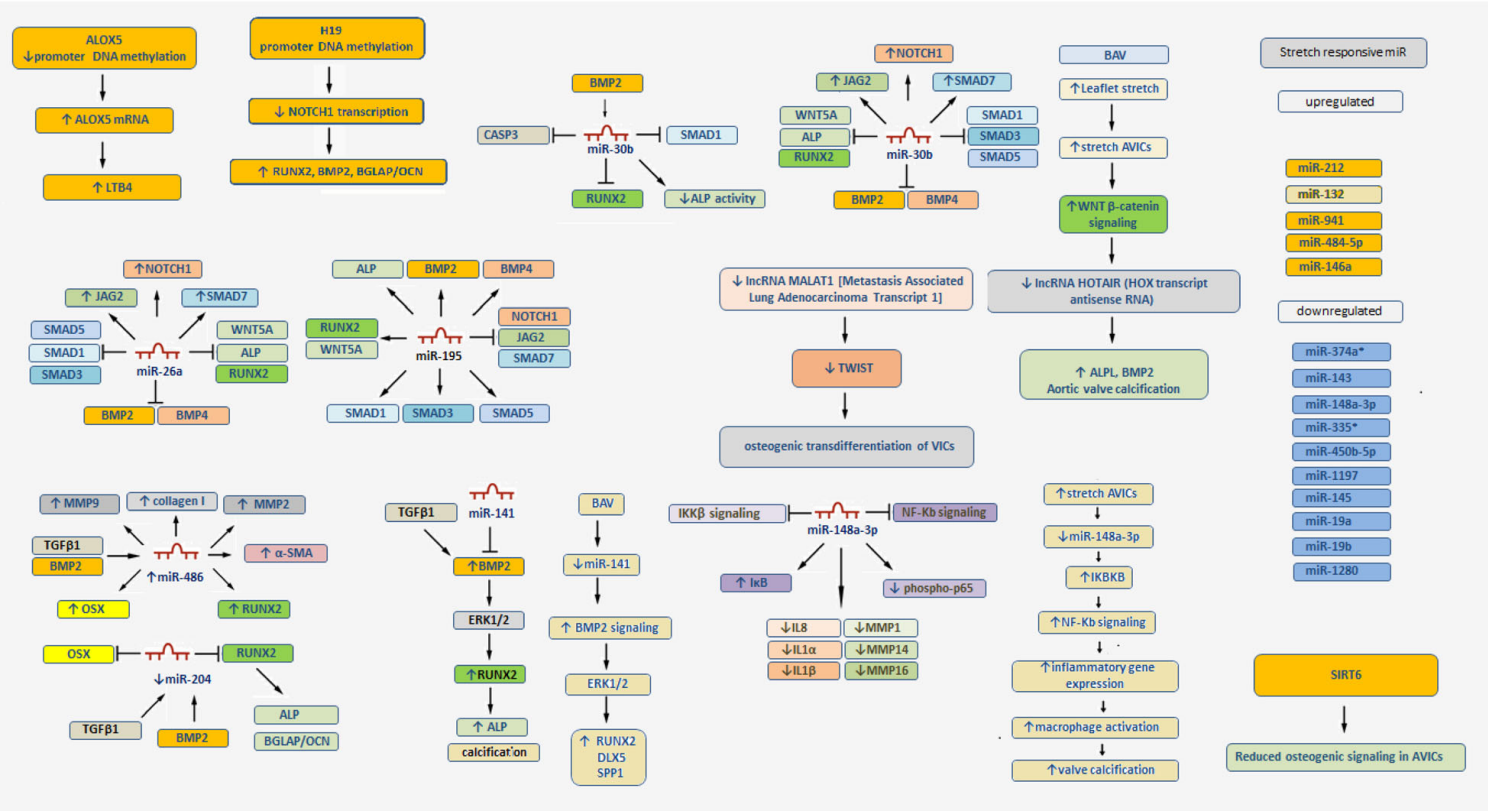
Epigenetic mechanisms currently associated with aortic VICs. *ALP* alkaline phosphatase, liver/bone/kidney, *ALOX5* arachidonate 5-lipoxygenase, *AVICs* aortic valve interstitial cells, *BAV* bicuspid aortic valve, *BGLAP/OCN* bone gamma-carboxyglutamate protein/osteocalcin, *BMP2* bone morphogenetic protein 2, *BMPR2* bone morphogenetic protein receptor type 2*, β-catenin* CTNNB1/catenin (cadherin-associated protein), beta 1, *CASP3* Caspase 3, apoptosis-related cysteine peptidase, *DLX5* distal-less homeobox 5, *DNMT3b* DNA (Cytosine-5-)-methyltransferase 3 beta, *ERK1* extracellular signal-regulated kinase 1/MAPK3 mitogen-activated protein kinase 3, *ERK2* extracellular signal-regulated kinase 2/MAPK1 mitogen-activated protein kinase 1, *H19* imprinted maternally expressed non-protein coding transcript, *IKKβ* inhibitor of kappa light polypeptide gene enhancer in b-cells, kinase beta, *NF-Kb* nuclear factor kappa b signaling pathway, *IGF-1* insulin-like growth factor 1, *ILα* interleukin alpha, *ILβ* interleukin beta, IL8 interleukin 8, *NOTCH1* notch homolog 1, translocation-associated (drosophila), *JAG2* jagged, *MMP1* matrix metallopeptidase 1, *MMP2* matrix metallopeptidase 2, *MMP9* matrix metallopeptidase 9, *MMP14* matrix metallopeptidase 14, *MMP16* matrix metallopeptidase 16, *OSX* SP7 transcription factor/osterix, *p65* RELA/RELA proto-oncogene, NF-KB subunit, *RUNX2* runt related transcription factor 2, *SMAD1* SMAD family member 1, *SMAD3* SMAD family member 3, *SMAD5* SMAD family member 5, *SMAD7* SMAD family member 7, *SPP1* secreted phosphoprotein 1/OPN osteopontin/BNSP bone sialoprotein I, *TGFβ1* transforming growth factor beta 1, *TGFBR2* transforming growth factor beta receptor 2, *WNT5a* Wnt family member 5a

Major epigenetic markers in human aortic VICs are presented in Fig. 2.

DNA methylation changes may also regulate gene expression pattern in aortic valve endothelial cells. Thus for example, White et al. reported *DNMT3B* as one of the genes affected by shear stress and decreased *NOTCH1* (Notch/drosophila/homolog 1/translocation-associated) signaling in primary human AVECs [63]. It is well known that *NOTCH1* regulates calcification related gene networks in human vascular and valvular endothelium [63]. Notably, NOTCH1 signaling is higher on the ventricular side, and it is considered protective for calcification [64, 65]. Furthermore, human *NOTCH1* mutations can cause both familial and sporadic AS disease [66].

Possible *NOTCH1*-dependent transcriptional and epigenetic mechanisms underlying this phenomenon are quite recently revealed by Theodoris et al. [67]. They used hiPSC (human-induced pluripotent stem cell)-derived ECs generated from two patients with nonsense mutations in *NOTCH1* and related unaffected individual to investigate how *NOTCH1* haploinsufficiency may cause aortic valve calcification [67].

Upon shear stress *NOTCH1* (+/−) ECs exhibited dysregulated epigenetic state and aberrant upregulation of pro-osteogenic, pro-oxidative and pro-inflammatory signaling pathways, thus recapitulating observed AVECs phenotype in AS [67]. Contrary, wild-type (WT; *NOTCH1+/+*) ECs cells under the same conditions suppressed these and activated homeostatic anti-atherogenic gene networks [67]. This was accompanied with corresponding alterations of DNA methylation marks, with so called ‘CpG Island shores’ (sequences up to 2 kb distant to CpG islands in promoter regions) showing the largest enrichment of methylation changes [67]. Their findings agree with previous reports of CpG shore regions displaying the most methylation differences in the context of specific tissue types regardless of species of origin [68].

Concludingly, all the above data, although some of them mostly inferential (osteoblastic transdifferentiation of VICs) and yet to be experimentally validated, strongly suggests that DNA methylation might be an important regulatory mechanism involved in the initiation and progression of AS.

#### Histone and chromatin marks

Emerging reports suggest that various HAT/HDAC (histone acetyltransferase/histone deacetylase) enzyme complexes have also an important roles in the patohistogenesis of AS.

For instance, Carter et al. have reported reduced mRNA and protein levels of class III histone deacetylase *SIRT1* (silent information regulator-two (1) in AS patients when compared to controls [69]. In addition, the levels of *SIRT1* expression were inversely correlated with transcriptional activity of *RETN* (resistin) gene in infiltrated macrophages, thus possibly contributing to the development of AS-associated inflammation [69]. Interestingly, Mohty et al. reported that higher resistin plasma levels in the elderly AS patients (≥ 70 years) are associated with lower LDL cholesterol and increased valvular calcification and inflammation compared to younger middle-aged patients [70]. This may well be associated with corresponding age-related changes in SIRT1 expression.

Roos et al. reported a significant age-related decrease of *SIRT1, −2, −3,-4, −6* and *SIRT7* expression in aortic valves of both *SOD2* (superoxide dismutase (2) haploinsufficient (*SOD2−/+*) and normal (*SOD2+/+*) mice and increased expression of *SIRT5* in aged, compared to young *SOD2+/−* mice [71]. They also reported markedly reduced expression of *SIRT6* in aortic valves from AS patients while knockout of *SIRT6* expression in mice resulted in a dramatic progeroid phenotype, with a much greater propensity for development of cardiovascular calcification and AS [72]. The same group showed that *SIRT6* inhibition promotes the formation of calcified nodules, induces amplification of pro-osteogenic signaling pathways and favors osteogenic differentiation of cultured mouse aortic VSMCs and VICs obtained from *SIRT6*-deficient mice, porcine, or human patients [73]. Also, *SIRT6*-deficient cells exhibited increased p-*SMAD1/5/8* levels while the global *SIRTs* inhibition resulted with increased histone acetylation and protein levels of osteogenic markers *SP7* and *RUNX2*. Furthermore, *SIRTs* inhibition has also enhanced the transcriptional responses of *SP7* and *RUNX2* gene in BMP2 treated VSMCs and VICs [73, 74].

Moreover, the data obtained in hypercholesterolemic (*LA*, *LDLR* ^*−/−*^ *APOB* ^*100/100*^) mice that were WT (*LA-SIRT6+/+*) or heterozygous for *SIRT6* (*LA-SIRT6+/−*) suggested the context-dependent role of *SIRT6* in hypercholesterolemia-induced valvular dysfunction, and modulation of hypertrophic responses to progressive increases in LV afterload [74–76]. Notably, 12-month-old *LA-SIRT6+/−* mice fed a western diet showed dramatically worsened aortic valve dysfunction and AS compared to age-matched WT or 3-month-old LA-*SIRT6+/−* mice that was also associated with reductions in LV ejection fraction [74]. In addition, reductions of *SIRT6* in the presence of chronic LV pressure overload significantly accelerated the rate of AS development in female but not male mice thus highlighting the biological influence of gender related factors on disease progression and AS-induced LV dysfunction [76].

The class I (HDAC1 and HDAC3) and class II (HDAC5, HDAC7, and HDAC9) HDAC family members were also found differentially expressed in human and porcine aortic valve cells [63, 77, 78]. For instance, HDAC1 was reported as shear-responsive transcripts thus suggesting its possible involvement in flow mediated regulation of side-specific gene expression in aortic valve endothelium [77]. In addition, *HDAC3, −7,* and −*9* transcripts levels have been found decreased in HOTAIR (HOX transcript antisense RNA) siRNA treated VICs cells while HDAC5 was detected as one of gene transcripts that were activated by flow and affected by decreased *NOTCH1* activity in human AVECs [63, 78].

It is well known that reduced *HDAC1* expression and its decreased recruitment to the promoters of osteoblast marker genes represent an important step for osteoblasts differentiation and vascular calcification [79, 80]. It has been also reported that HDAC3 physically interacts with *RUNX2* thus suppressing its transcriptional activity [81] Furthermore, dysregulation of HDAC3 mediated epigenetic silencing of *TGFβ1* (transforming growth factor beta 1) is required during valve development to maintain VICs quiescence and may represent a predisposing factor for development of BAV and other congenital heart diseases [82]. In addition, HDAC5 is involved in RUNX2 degradation while HDAC7 represses its activity during osteoblastogenesis in deacetylase independent manner [82–85]. Furthermore, since HDAC7 suppresses the expression of *MMP10*, its downregulation observed in VICs may play a role in biomechanical and HOTAIR lncRNA dependent upregulation of procalcific and inflammatory genes related to BAV induced AS [78, 86, 87]. Nevertheless, despite all of the above data, the exact role, if any, of these HDAC enzymes in development and progression of AS has yet to be experimentally validated.

The importance of histone code alterations in the development of AS was also reinforced by previously mentioned analysis of hiPSC-derived ECs reported by Theodoris et al. [67]. They showed a clear relationship between the expression of oxidative, inflammatory and calcification related genes in WT (*NOTCH1+/+*) and haploinsufficient (*NOTH1+/−*) ECs mediated by fluid-flow conditions and *NOTCH1*-dependent distribution of activated (H3K4me3, H3K27ac, and H3K4me1) and repressive (H3K27me3) histone marks [67]. More specifically, the shear stress response in WT ECs resulted in activation of anti-osteogenic and anti-oxidation genes and repression of proinflammatory loci that was correlated with the occupancy of their enhancers by repressive H3K27me3 or active H3K27ac histone marks [67]. Quite contrary, upon shear stress *Notch1+/−* ECs exhibited aberrant upregulation of pro-osteogenic genes associated with increased H3K4me3, H3K27ac, and H3K4me1 histone marks while H3K27ac marks were increased in active enhancers of pro-inflammatory genes under both static and shear flow conditions [67]. Moreover, anti-calcific and anti-atherogenic genes downregulated by *NOTCH1* haploinsufficiency showed decreased H3K27ac active enhancer marks as well as increased repressive H3K27me3 marks [67]. Importantly, alterations of histone code were in most cases accompanied with corresponding changes of DNA methylation marks [67]. More specifically, the regions hypermethylated in *Notch1* haploinsufficient ECs lost H3K4me3 or H3K27ac activating marks present in WT ECs while regions hypomethylated in *N1+/−* ECs gained H3K4me3 or H3K27ac marks [67].

Although it remains to be determined whether the used hiPSC-derived ECs accurately model molecular pathways induced in diseased human AVECs, these data presented by Theodoris et al. represent a significant step forward in deciphering the complexity and mutual interconnection of epigenome alterations underlying the pathogenesis of AS.

Other genes involved in regulation of epigenetic histone marks and/or chromatin remodeling that were found differentially expressed in stenotic aortic valves are presented in Table 1. Given the important role the mechanical forces, proatherogenic risk factors and intrinsic (epi)genetic variants, as well as, age and gender are playing in disease onset and progression, the known transcriptional profile of corresponding histone and chromatin remodeling factors induced by them in experimentally modified aortic valve endothelial and interstitial cells are presented as well. Specified genes are selected based on reanalysis of published data using *DAVID (*database for annotation visualization and integrated discovery), *EpiFactors* (database for epigenetic factors, corresponding genes and products) and *Genecard* (the human gene database) free online databases [88–91].

**Table 1:** Genes involved in regulation of epigenetic histone marks and/or chromatin remodeling

*G*ene name; official gene symbol	Epigenetic function	Expression in stenotic valves and aortic valve cells [reference]
AT-rich interaction domain 1A; SWI/SNF-related, matrix-associated, actin-dependent regulator of chromatin subfamily F Member 1; ***ARID1A***	Chromatin remodeling cofactor	↑ PAVECs* [177]
AT-rich interaction domain 4B; ***ARID4B***	HAT cofactor	↑ HAVICs^1^ [78]
AT-rich interaction domain 5B; ***ARID5B***	HDM cofactor (H3K9me2 → H3K9)	↑ AS [99], ↑HAVICs^1^ [78], ↑ PAVICs^#^ [178], (NOTH1+/−) ECs^a^ [67]
*Anti-silencing function 1B histone chaperone;* ***ASF1B***	Histone (H3/H4) chaperone	HAVECs^b^ [63], HAVECs^c^ [77]
ASH2 like histone lysine methyltransferase complex subunit; ***ASH2L***	HMT cofactor, component of COMPASS H3K4 methyltransferase complex	HAVECs^c^ [77], ↑ PAVICs^#^ [178], ↓ HAVECs by miR-483p and unidirectional shear stress [179]
ATRX, chromatin remodeler; ***ATRX***	Chromatin remodeling	↑ HAVICs^1^ [78]
BTG3 associated nuclear protein; ***BANP***	Histone acetylation	HAVECs^c^ [77]
BRCA1 associated protein 1; ***BAP1/*** *KIAA0272*	Deubiquitination (H2AK119ub1 → H2AK119), PcG protein;	↑ PAVECs* [177]
BRCA1 Associated RING Domain 1; ***BARD1***	Histone ubiquitination (H2AX, H2A, H2B, H3, H4 → H2AXub, H2Aub, H2Bub, H3ub, H4ub)	↓ PAVECs* [177]
Bromodomain adjacent to zinc finger domain 1A; ***BAZ1A***/*ACF1*	Histone chaperone	HAVECs^c^ [77]
BCL6 corepressor; ***BCOR***	PcG protein	↑ AS [99]
BMI1 proto-oncogene, polycomb ring finger; ***BMI1***	PcG protein	HAVECs^b^ [63]
Bromodomain containing 1; ***BRD1***	Histone acetyl-lysine reader	↓ HAVICs^1^ [78]
Bromodomain containing 9; ***BRD9***	Histone acetyl-lysine reader	↓ HAVICs^1^ [78]
Brain and reproductive organ-expressed; TNFRSF1A modulator; ***BRE***	Histone [H2A(X)] ubiquitination cofactor	↓ HAVICs^1^ [78], ↓ PAVECs* [177]
Bromodomain and PHD finger containing 1; ***BRPF1***	Histone acetyl-lysine reader, component of the MOZ(KAT6A/MYST3)/MORF(KAT6B/MYST4) HAT complex	HAVECs^b^ [63], ↓ PAVICs** [180]
Bromodomain and WD repeat domain containing 1; ***BRWD1***	Histone acetyl-lysine reader; chromatin remodeling	↑ HAVICs^1^ [78]
Coactivator associated arginine methyltransferase 1; ***CARM1***/PRMT4	Histone-Arginine(R) methyltransferase (H3R17 → H3R17me, H3R17me2a)	HAVECs^b^ [63], ↓ HAVICs^1^ [78]
Chromobox 2; ***CBX2***	Methyl-lysine(K) reader (H3K9me3, H3K27me3), component of PRC1-like complex	(NOTH1+/−) ECs^a^ [67]
Chromobox 3; ***CBX3***	Methyl-lysine(K) reader (H3K9me3), epigenetic repressor- interacts with MECP2 and modulates epigenetic gene silencing during myogenic differentiation	↑ AS [99], cardiomyocytes of AS patients with cardiomyopathy [181]
Chromobox 4; ***CBX4***	Methyl-lysine(K) reader (H3K9me3), component of PRC1-like complex	↑ BAVc vs. TAVn [182]
Chromobox 7; ***CBX7***	Methyl-lysine(K) reader (H3K9me3, H3K27me3), component of PRC1-like complex	↓ HAVICs^1^ [78]
Chromobox 8; ***CBX8***	Methyl-lysine(K) reader (H3K9me3, H3K27me3), component of PRC1-like complex	↑ BAVc vs. TAVn, ↑ TAVc vs. TAVn [182]
CECR2, histone acetyl-lysine reader; ***CECR2***	Histone Acetyl-Lysine(K) reader, component of CERF SWI/SNF chromatin remodeling complex	↓ AS [99], ↓ BAVc vs. TAVn, ↓ TAVc vs. TAVn [182], ↑ BAVr vs. BAVc [183]
Chromatin assembly factor 1 subunit A; ***CHAF1A***	Histone chaperone and epigenetic regulator, primary component of CAF1	HAVECs^b^ [63]
Chromatin assembly factor 1 subunit B; ***CHAF1B***	Histone chaperone and epigenetic regulator, primary component of CAF1 complex	HAVECs^b^ [63]
Chromodomain helicase DNA binding protein 1; ***CHD1***	Chromatin remodeling factor	↓ PAVECs* [177]
Chromodomain helicase DNA binding protein 1; ***CHD1L***	Chromatin-remodeling following DNA damage, interacts with poly(ADP-ribose) and catalyzes PARP1-stimulated nucleosome sliding	↓ HAVICs^1^ [78]
Chromodomain helicase DNA binding protein 9; ***CHD9***	Chromatin related mesenchymal modulator, associates with A/T-rich regulatory regions in promoters of genes that participate in the differentiation of progenitors during osteogenesis	HAVECs^c^ [77]
CREB binding protein; ***CREBBP***	CREB and its paralog p300 (EP300) constitute the KAT3 family of HATs in mammals	↑ HAVICs^1^ [78]
CXXC finger protein 1; ***CXXC1***	Binds DNA sequences with unmethylated CpG, epigenetic regulator of both cytosine and histone methylation, component of COMPASS/SETD1A/B HMT complex	↓ HAVICs^1^ [78]
DEK proto-oncogene; ***DEK***	Chromatin remodeling, histone chaperone	↓ PAVECs* [177]
Double PHD fingers 3; ***DPF3***	Histone acetylation and methylation reader of BAF chromatin remodeling complex, recruits BRG1 to genomic targets	HAVECs^b^ [63], ↑ hypertrophic hearts of AS patients [96]
EMSY, BRCA2 interacting transcriptional repressor; ***EMSY*** */C11orf30*	Histone methylation cofactor, part of EMSY/KDM5A/SIN3B HMT complex	↓ PAVICs^#^ [178]
Enhancer of polycomb homolog 1; ***EPC1***	PcG protein, component of the NUA4 HAT complex	↑ PAVECs* [177]
Enhancer of Zeste 2 Polycomb Repressive Complex 2 Subunit; ***EZH2*** */KMT6*	PcG protein, Histone methylation (H3K27 → H3K27me1, H3K27me2, H3K27me3)	↓ PAVECs* [177]
EYA transcriptional coactivator and phosphatase 1; ***EYA1***	Dephosphorylation of Tyr(Y)-142 in H2AX (H2AXY142ph)	↓ AS [99], ↑ BAVc vs. TAV; ↑ BAVr vs. TAV [183]
EYA transcriptional coactivator and phosphatase 4; ***EYA4***	Dephosphorylation of Tyr(Y)-142 in H2AX (H2AXY142ph)	HAVECs^b^ [63], ↓ BAVc vs. TAVn [182]
Histone deacetylase 1; ***HDAC1***	Class I HDAC member	HAVECs^c^ [77], ↓ PAVECs* [177]
Histone deacetylase 3; ***HDAC3***	Class I HDAC member	↓ HAVICs^1^ [78]
Histone deacetylase 5; ***HDAC5***	Class II HDAC member	HAVECs^b^ [63], ↑ PAVECs* [177]
Histone deacetylase 7; ***HDAC7***	Class IIa HDAC member	↓ HAVICs^1^ [78]
Histone deacetylase 9; ***HDAC9***	Class II HDAC member	↓ HAVICs^1^ [78]
Histone cell cycle regulator; ***HIRA***	Histone chaperone, cooperates with ASF1A to promote replication-independent chromatin assembly, required for early steps of osteoblastic differentiation, interacts with OGT and regulates nucleosome assembly and cellular senescence.	HAVECs^b^ [63], ↓ AS [99]
Helicase like transcription factor; SWI/SNF Related, Matrix Associated, Actin Dependent Regulator of Chromatin, Subfamily A, Member 3; ***HLTF***	Chromatin remodeling cofactor, member of the SWI/SNF family, acts as a ubiquitin ligase for ‘Lys-63’-linked polyubiquitination of chromatin-bound PCNA.	Blood dried spots- congenital AS [42]
IKAROS family zinc finger 1; ***IKZF1***	Chromatin remodeling	↑ BAVc vs. TAVn, ↑ TAVc vs. TAVn [182]
Inhibitor of growth family member 3; ***ING3***	Chromatin remodeling, HAT cofactor, component of the NUA4 HAT complex, binds H3K4me3 histone marks	↑ HAVICs^1^ [78], ↓ AS [88], ↓ PAVECs* [177]
INO80 complex subunit C; ***INO80C***	Chromatin remodeling cofactor, component of the INO80 chromatin remodeling complex	↑ HAVICs^1^ [78]
Janus Kinase 2; ***JAK2***	Phosphorylation of Tyr(Y)-41 of histone H3 (H3T41 → H3Y41ph)	↑ rat AVICs*** [64]
Jade family PHD finger 1; ***JADE1*** */PHF17*	Histone acetylation (H3, H4 → H3ac, H4ac), component of HBO1 HAT complex	↓ AS [99]
*Jade Family PHD Finger 2;* ***JADE2***	Histone acetylation (H3, H4 → H3ac, H4ac), component of HBO1 HAT complex	HAVECs^c^ [77]
Arginine demethylase and lysine hydroxylase; ***JMJD6***	Histone arginine demethylase (H3R2me, H4R3me → H3R2, H4R3) and a lysyl-hydroxylase.	↑ PAVICs^#^ [178]
Lysine acetyltransferase 2A; ***KAT2A*** */GCN5*	HAT	HAVECs^c^ [77], ↓ PAVECs* [177]
Lysine acetyltransferase 2B; ***KAT2B***	HAT	HAVECs^b^ [63], ↑ HAVICs^1^ [78]
Lysine acetyltransferase 6A; ***KAT6A*** */MYST3*	HAT (H3, H4 → H3ac, H4ac), component of MOZ/MORF HAT complex	↓ PAVECs* [177]
Lysine acetyltransferase 7; ***KAT7*** */HBOA/MYST2*	HAT (H4 → H4ac), component of HBO1 HAT complex	↓ PAVECs* [177]
Lysine acetyltransferase 8; ***KAT8*** */MYST1/MOF*	HAT (H2A, H3, H4 → H2Aac, H3ac, H4ac), member of the MYST HAT family	HAVECs^b^ [63]
Lysine demethylase 1A; ***KDM1A*** */ KIAA0601*	Histone demethylase (H3K4me1, H3K4me2, H3K9me → H3K4, H3K9), component of NuRD complex	↓ PAVECs* [177]
Lysine demethylase 3A; ***KDM3A/*** *JMJD1*	Histone demethylase (H3K9me1, H3K9me2 → H3K9)	HAVECs^b^ [63]
Lysine demethylase 5C; ***KDM5C*** */Xe169/ JARID1C*	Histone demethylase (H3K4me3 → H3K4me2, H3K4me1)	↑ PAVICs** [180], ↓ PAVECs* [177]
Lysine demethylase 6B; ***KDM6B*** */JMJD3*	Histone demethylases (H3K27me2. H3K27me4 → H3K28), promotes osteogenic differentiation of human MSCs, regulates osteoblast differentiation via transcription factors RUNX2 and SP/OSX	↑ PAVICs^#^ [178]
Lysine demethylase 7A; ***KDM7A*** */JHDM1D*	Histone demethylase (H3K9me2, H3K27me2, H4K20me1 → H3K9, H3K27, H4K20)	HAVECs^b^ [63]
Lysine methyltransferase 2A; ***KMT2A*** */MLL*	HMT (H3K4 → H3K4me), catalytic subunit of MLL1/MLL complex	↓ PAVECs* [177], ↓ AS [99]
Lysine methyltransferase 2B; ***KMT2B*** */KIAA0304*	HMT (H3K4 → H3K4me3)	↓ PAVECs* [177]
Lysine methyltransferase 2E; ***KMT2E***	HMT (H3K4 → H3K4me1, H3K4me2)	↓ PAVICs^#^ [178]
L(3)Mbt-Like 3 (Drosophila); ***L3MBTL3*** */KIAA1798*	Methyl-lysine(K) reader (H4K20me), putative PcG protein	HAVECs^c^ [77], ↑ HAVICs^1^ [78], ↑ PAVECs vs. PAEC* [177]
Lysyl oxidase like 2; ***LOXL2***	Counteracts HMTs, acts as H3K4me2/3 deaminase, thus giving cells an additional method for removing methylated residues	↑ AS [99], ↑ BAVc vs. TAVn, ↑ TAVc vs. TAVn [182]
Leucine rich repeats and WD repeat domain containing 1; ***LRWD1***	Chromatin remodeling, binds H3K9me3, H3K20me3 and H4K27me3 in a cooperative manner with DNA methylation	↓ HAVICs^1^ [78]
MYC associated factor X; ***MAX***	Histone modification write cofactor involved in histone methylation and acetylation, epigenetic sensor of 5-carboxylcytosine	↓ PAVICs** [180], ↑ PAVECs vs. PAEC^$^ [177]
Microspherule protein 1; ***MCRS1***	Putative regulatory component of INO80 chromatin remodeling complex with HAT activity (H4K5, H4K8, H4K16 → H4K5ac, H4K8ac, H4K16ac)	↓ HAVICs^1^ [78], ↓ PAVECs vs. PAEC^$^ [177]
Megakaryoblastic Leukemia (Translocation); ***MKL1/*** *MRTFA*	Epigenetic orchestrator that connects chromatin and histone modification to: oxidative stress and oxLDL-induced endothelial injury, LPS and endothelin induced proinflammatory gene expression in macrophages and VSMCs, correspondingly; expression of SMC differentiation markers; TGFβ-induced fibrogenesis; cardiac hypertrophy	↑ AS [184]
Nucleosome assembly protein 1 like 2; ***NAP1L2***	Histone modification cofactor; associated with histone H3 and H4 acetylation involved in nucleosome assembly and exchange of H2A-H2B dimmers	HAVECs^b^ [63], ↓ AS [88], ↓ BAVc vs. TAVn, ↓ TAVc vs. TAVn [182]
Nuclear autoantigenic sperm protein; ***NASP***	Histone chaperone, chromatin remodeling	HAVECs^b^ [63], HAVECs^c^ [77]
Nuclear receptor corepressor 2; ***NCOR2***	Histone acetylation eraser	HAVECs^b^ [63], ↑ HAVICs^1^ [78], (NOTH1+/−) ECs^a^ [67]
O-linked N-acetylglucosamine; GlcNAc transferase; ***OGT***	O-GlcNAc transferase, PcG protein, modifies members of the TET family	HAVECs^c^ [77], ↑ HAVICs^1^ [78], ↑ PAVECs* [177], LV tissue from AS patients [185], ↓ HAVECs by miRNA-181b and oscillatory shear stress [186]
Poly; ADP-ribose polymerase 1; ***PARP1***	Chromatin remodeling; Histone H1 poly[ADP]-ribosylation, modulates chromatin architecture in a context-dependent manner, controls epigenetic modifications of both histones and DNA, poly(ADP-ribosyl)ation (PARylation)--participates in the establishment and maintenance of a genome methylation pattern	↑ PAVECs vs. PAEC* [177], ↑ TAVc vs. BAVc, leukotriene C4(LTC4) treated VICs [187]
PAX3 and PAX7 binding protein 1; ***PAXBP1*** */C21orf66*	Adapter protein linking the transcription factors PAX3 and PAX7 to the histone methylation machinery, involved in myogenesis.	↓ AS [184], ↑ HAVICs^1^ [78]
PAX interacting protein 1; ***PAXIP1***	HMT cofactor (H3K4 → H3K4me3), subunit of the MLL3/MLL4 HMT complex	↓ PAVECs* [177]
Polycomb group ring finger 2; ***PCGF2*** */MEL18*	PcG protein, component of PRC1-like complex	↑ PAVECs* [177]
Polycomb group ring finger 5; ***PCGF5***	PcG protein, component of PRC1-like complex	↑ AS [114]
PHD finger protein 1; ***PHF1***	PcG protein, component of PRC2 complex	↓ HAVICs^1^ [78], ↑ PAVECs vs. PAECs* [177]
PHD finger protein 2; Jumonji C domain-containing histone demethylase 1E; ***PHF2*** */GRC5*	Lysine(K) demethylase (H3K9me2 → H3K9), component of PKA-dependent PHF2-ARID5B HDM complex	↑ PAVECs vs. PAECs*^$^ [177]
PHD finger protein 19; ***PHF19***	PcG protein, chromatin remodeling, HAT cofactor, binds H3K36me3 and recruits the PRC2 complex	↓ HAVICs^1^ [78]
Protein Kinase N1; ***PKN1***	Histone phosphorylation at threonine(T)11 (H3T11 → H3T11ph)	↓ HAVICs^1^ [78]
PR/SET domain 1; ***PRDM1***	HMT cofactor (H3K9 → H3K9me), lack intrinsic HMT activity, but instead recruits G9A/EHMT2/H3K9 HMT	HAVECs^b^ [63], (NOTH1+/−) ECs^a^ [67], HAVECs^c^ [77], ↑ HAVICs^1^ [78]
PR/SET domain 4; ***PRDM4***	Histone arginine methylation (H4R3 → H4R3me2s)	↓ PAVECs* [177]
PR/SET domain 6; ***PRDM6*** */PRISM*	HMT cofactor (H3R2, H4K20 H3R2me1, H3R2me2, H4K20me1), acts as a transcriptional repressor of VSMCs gene expression, lack intrinsic HMT activity, but instead recruits G9A/EHMT2/H3K9 HMT	↓ PAVICs** [180]
PR/SET domain 8; ***PRDM8***	HMT preferentially acting on H3K9	HAVECs^b^ [63]
Protein arginine methyltransferase 1; ***PRMT1***	Arginine(R) methyltransferase (H4R3 → H4R3me1, H4R3me2a), participate in reading of repressive DNA methylation marks	HAVECs^c^ [77], ↓ HAVICs^1^ [78]
Protein arginine methyltransferase 5; ***PRMT5***	Arginine(R) methyltransferase (H3R8, H4R3 → H3R8me, H4R3me)	↓ HAVICs^1^ [78]
RB binding protein 4, chromatin remodeling factor; ***RBBP4***	Histone chaperone, part of the Mi-2/NuRD chromatin remodeling complex	↑ HAVICs^1^ [78], ↓ PAVECs* [177]
RB binding protein 5, histone lysine methyltransferase complex subunit; ***RBBP5***	HMT cofactor (H3K4 → H3K4me1, H3K4me2, H3K4me3), part of the COMPASS and MLL1/MLL complex	↑ HAVICs^1^ [78]
RB transcriptional corepressor like 1; ***RBL1***	Recruits and targets histone methyltransferases KMT5B and KMT5C, leading to epigenetic transcriptional repression, controls histone H4 Lys-20 trimethylation.	↓ PAVECs* [177]
RB transcriptional corepressor like 2; ***RBL2***	Chromatin remodeling, repression of DNMTs (e.g. DNMT3A, DNMT3B) and control of global DNA methylation,	↓ HAVICs^1^ [78], ↓ AS [114]
RuvB like AAA ATPase 1; ***RUVBL1***	Chromatin remodeling, Histone phosphorylation, component of the NuA4 and INO80 complex	↓ HAVICs^1^ [78], ↓ PAVECs* [177]
SAM domain, SH3 domain and nuclear localization signals 1; ***SAMSN1***	Implicated in the epigenetic control of gene expression, regulates the activity of HDAC1	↑ AS [99], ↑ AS [114], ↑ TAVc vs. TAVn [182]
SATB homeobox 1; ***SATB1***	Chromatin remodeling cofactor	HAVECs^b^ [63]
SET domain bifurcated 1; ***SETDB1***	HAT (H3K9 → H3K9me3	↑ PAVICs^#^ [178]
SET domain and mariner transposase fusion gene; ***SETMAR***	HAT (H3K4, H3K36 → H3K4me, H3K36me)	↑ HAVICs^1^ [78]
SWI/SNF related, matrix associated, actin dependent regulator of chromatin, subfamily a, member 1; ***SMARCAL1***	Chromatin remodeling	↑ PAVICs^#^ [178], ↓ PAVECs vs. PAEC^$^ [177]
SWI/SNF related, matrix associated, actin dependent regulator of chromatin, subfamily a, member 2; ***SMARCA2***	Chromatin remodeling, histone modification reader (targets H3)	↑ HAVICs^1^ [78]
SWI/SNF related, matrix associated, actin dependent regulator of chromatin, subfamily b, member 1; ***SMARCB1***	core subunit of the SWI/SNF (BAF) chromatin-remodeling complex, histone modification reader (targets H3K56)	↓ HAVICs^1^ [78], ↓ PAVECs* [177]
SWI/SNF related, matrix associated, actin dependent regulator of chromatin subfamily c member 1; ***SMARCC1***	Chromatin remodeling cofactor	HAVECs^c^ [77]
SWI/SNF related, matrix associated, actin dependent regulator of chromatin subfamily c member 2; ***SMARCC2***	Chromatin remodeling cofactor	↑ PAVECs* [177]
SMYD family member 5; ***SMYD5***	HMT (H4K20 → H4K20me3), part of NCoR complex	↓ HAVICs^1^ [78]
Suppressor of variegation 3–9 homolog 2; ***SUV39H2*** */KMT1B*	HAT (H3K9me1 → H3K9me3)	HAVECs^b^ [63]
Transcriptional adaptor 3; ***TADA3***	HAT cofactor, component of the PCAF complex	↓ HAVICs^1^ [78]
TATA-box binding protein associated factor 5 like; ***TAF5L***	HAT cofactor, component of the PCAF complex	↓ HAVICs^1^ [78]
Tripartite motif containing 24; ***TRIM24***	Human chromatin reader, lysine acetylated histone binding	↑ HAVICs^1^ [78]
Tripartite motif containing 28; ***TRIM28***	Histone modification reader (targets H3)	↓ HAVICs^1^ [78]
Ubinuclein 1; ***UBN1***	HMT cofactor	HAVECs^c^ [77]
Ubiquitin protein ligase E3 component n-recognin 2; ***UBR2*** */KIAA0349*	Modification Histone ubiquitination (targets H2A)	↓ PAVECs* [177]
Wolf-Hirschhorn syndrome candidate 1; ***WHSC1*** */NSD2*	HMT (H3K27 → H3K27me)	HAVECs^b^ [63], (NOTH1+/−) ECs^a^ [67]
Wolf-Hirschhorn syndrome candidate 1-like 1; ***WHSC1L1*** */NSD3*	member of the NSD methyltransferase family (targets H3K4, H3K27)	↑ HAVICs^1^ [78]

Legend: AS- aortic valve stenosis; ASF1A- anti-silencing function 1a histone chaperone; BAVc- calcified stenotic bicuspid aortic valve; BAVr- bicuspid aortic valve with redundant leaflets and/or minimal calcification; BRG1- SWI/SNF related, matrix associated, actin dependent regulator of chromatin, subfamily a, member 4; DNMTs- DNA methyltransferase; HAT-histone acetyltransferase; HAVECs- human aortic valve endothelial cells; HAVICs- human aortic valve interstitial cells; HDM- histone demethylase; HMT- histone methyltransferase; (NOTH1+/−) ECs- hiPSC (human induced pluripotent stem cell)-derived endothelial cells generated from two patients with nonsense mutations in NOTCH1; MECP2- methyl CpG binding protein 2; CAF1-; oxLDL- oxidized low-density lipoprotein; LPS- lipopolysaccharide; PAECs- porcine aortic endothelial cells; PAVECs- porcine aortic valve endothelial cells; PAX3- paired box 3; PAX7- paired box 7; PcG- polycomb group protein; PCNA- proliferating cell nuclear antigen; RAVICs- rat aortic valve interstitial cells; RUNX2 – runt related transcription factor 2; SP7/OSX- osterix; SMC -smooth muscle cell; TAVc- calcified stenotic tricuspid aortic valve; TAVn -noncalcified tricuspid aortic valve, without stenosis; TGFβ- transforming growth factor beta; VSMCs- vascular smooth muscle cells

^1^Genes altered > 1.2-fold with a *p* < 0.05 in the HOTAIR siRNA microarray data; ^a^NOTCH1 haploinsufficiency, shear or static flow significant; ^b^affected by flow and decreased NOTCH1; ^c^shear-sensitive transcripts; *shear stress conditions modeling laminar flow; ^**^male vs. female; ^***^ inhibition of NOTCH1 signaling; ^#^ 2 years old vs. Juvenile Rapacz familial hypercholesterolemic (RFH) swine. ﻿NOTE: Official gene symbols are presented in bold and italicized, aliases are presented in italics﻿

#### Histone/chromatin alteration in AS-induced LVH

Alteration of histone marks and chromatin remodeling might also contribute to the pathological response of LV myocardium to increased afterload in AS, thus leading to CH and HF [92]. Although the initial hypertrophic responses seem to be an adaptation to those stimuli, the sustained stress may lead to reactivation of fetal genes, which is possible due to the interplay of transcription factors, HAT/ADAC and ATP-dependent chromatin remodeling complexes [92].

Thus, for example, Bovil et al. reported the reduced expression of *JARID2* (Jumonji and AT-rich interaction domain containing 2) by mechanical stress in LV biopsy samples from AS patients [93]. They showed that *JARID2* regulates the transcription of *ANF* (atrial natriuretic factor), *MYL7* (myosin light chain 7), and *MYH2* (myosin heavy chain 2), and contributes to reexpression of the fetal gene program in cardiomyocytes subjected to increased afterload [93]. More specifically, the reduced *JARID2* expression contributes to increased *ANF* and *MYL7* and decreased *MYH2* expression [93]. In addition, Sanulli et al. reported that *JARID2* interacts with *PRC2* (Polycomb repressive complex 2) responsible for methylation of histone H3 lysine 27 (H3K27me2/3) through its EZH (enhancer of zeste; *EZH1/2*) members and plays an essential role in regulating gene expression during embryonic development [94]. This *JARID2* mediated recruitment of the PRC2 complex to target genes is also required for proper expression of *NOTCH1* [95].

Very recently, Cui et al. reported a significant upregulation of *DPF3/BAF45C* [Double PHD Fingers 3/*BRG1*-Associated Factor 45C, a histone acetylation and methylation reader of the SWI/SNF (SWItch/Sucrose Non-Fermentable)-like ATP-dependent BAF (BRG/BRM-associated factor) chromatin remodeling complex] and its two individual splice variants DPF3a (BAF45c1) and DPF3b (BAF45c2) in hypertrophic hearts of patients with hypertrophic cardiomyopathy or AS [96]. Importantly, DPF3a is expressed as a fetal-like gene, whose activation by *CSNK2* (casein kinase 2; phosphorylates DPF3a at serine 348) upon hypertrophic stimuli switches cardiac fetal gene expression from being silenced by HEY (the hairy and enhancer of split-related family of basic helix-loop-helix /bHLH/ transcription factors; HEY1/2 and HEYL) proteins to being re-activated by *BRG1/SMARCA4* (brahma-related gene 1; SWI/SNF related, matrix associated, actin-dependent regulator of chromatin, subfamily a, member 4) [96]. Consequently, the transcription of downstream targets such as *NPPA* (natriuretic peptide A), *FOXO1* (Forkhead box O1), *GATA4* (growth arrest and DNA damage-inducible protein 45; mediates active DNA demethylation pathways), *TBX3* (T-box 3) and *SMAD7* (SMAD family member 7) were found significantly upregulated, thus promoting cardiac hypertrophy [96].

As already known, *BRG1* is highly expressed in the developing heart while *BRM/SMARCA2* (Brahma; SWI/SNF related, matrix associated, actin dependent regulator of chromatin, subfamily a, member 2) represents the prominent catalytic unit of BAF chromatin remodeling complex in adult cardiomyocytes [96]. However, upon hypertrophic stimuli adult cardiomyocytes switch to a fetal-like state and *BRG1* is reactivated and with its embryonic partners (HDACs and poly (ADP ribose) polymerases /PARPs/) forms *BRG1-HDAC-PARP* chromatin remodeling complex to induce a pathological gene expression [96].

The importance of *BRG1* for aortic valve development was also previously reported by Akerberg et al. [97]. They showed that endocardial *BRG1*-deficient embryos develop thickened aortic valves that are frequently bicuspid and progress to a myxomatous, disease-like state but does not become overtly calcified [97].

Taken together, these data provide new insight into the complexity of epigenetic gene regulation in diseased aortic valve cells and affected myocardium. Without any doubt, we currently see only the very tip of histone and chromatin alterations lying under the surface of AS patohistogenesis with numerous new players expected to emerge.

#### LncRNAs

Long noncoding RNAs are also emerging as powerful epigenetic regulators of AS. The recent comparative transcriptome analysis of calcified aortic valves and 32 other human tissues reported by Wang et al. revealed a total of 725 aortic valve-specific lncRNAs [98]. However, the exact role of lncRNA transcripts in patohistogenesis of AS is still poorly known and limited to few examples.

We already mentioned the role of H19 in osteogenic transdifferentiation of AVICs reported by Hadji et al. [46]. They also detected three upregulated (*AFAP1-AS1, CCND1* ncRNA, and *PRINS*) and five downregulated (*AK082072*, *APO-AS1*, *OIP5-AS1*, *PTENP1-AS*, and *SOX2-OT*) lncRNAs in calcific aortic valves when compared to control [46]. It is known that the exon 1 of *H19* harbors a primary miRNA sequence, which generates miR-675-3p and miR-675-5p, and miR-675-5p was found to directly target *H19* and counteracted osteoblast differentiation. However, Hadji et al. did not detect the expression of miR-675 neither in control or calcified aortic valves [46].

Previously, Carrion et al. have reported decreased transcript levels of *HOTAIR* (HOX transcript antisense RNA) in human BAVs as compared to the normal TAVs and in VICs exposed to cyclic stretch [78]. They also detected significant upregulation of several genes associated with calcification of VICs subjected to cyclic stretch (*ALP* and *BMP2*) and HOTAIR siRNA treatment (*ALP*, *BMP 1/4/6*, bone morphogenic protein receptor type 2/*BMPR2*, endothelin 1/*EDN1*, periostin/*POSTN*, *SOST*, matrix γ-carboxyglutamate (Gla) protein/*MGP*, and *MMP2/10/12*) [78]. Furthermore, the mechanoresponsive *HOTAIR* expression and epigenetic regulation (recruitment of PRC2 complexes which mediate trimethylation of lysine 27 on histone H3/H3K27me3 to *ALP* and *BMP2* promoters) of calcification related genes were significantly repressed by WNT β-catenin signaling pathway [78]. Interestingly HOTAIR siRNA treatment of cultured VICs resulted with decreased expression of *DNMT3B* and altered expression of number of genes involved in histone and chromatin modification (Table 1) and genes encoding various miR (miR-1228, miR-1978 and miR-586) and lncRNA transcripts (Table 2).

**Table 2:** LncRNA transcripts differentially expressed in stenotic aortic valves and experimentally modified aortic valve cells

LNCRNA name; symbol	Expression in stenotic valves and aortic valve cells [reference]
Psoriasis associated non-protein coding RNA induced by stress; ***PRINS/*** *NCRNA00074* AFAP1 Antisense RNA 1; ***AFAP1-AS1/*** *MGC10981* ***CCND1 associated ncRNA***	↑ AS [46]
MIR155 Host Gene; ***MIR155HG*** **/** *BIC (B-cell receptor inducible)/ NCRNA00172* Long intergenic non-protein coding RNA 467; ***LINC00607***/***LOC646324*** RUNX1 Intronic Transcript 1; ***RUNX1-IT1/*** *C21orf96*	↑ AS [99]
Imprinted maternally expressed transcript (Non-Protein Coding); ***H19*** **/** *LINC00008*	↑ AS [99] ↓ HAVECs^c^ (FO/VL; FO/FL; VO/VL) [77] (NOTH1+/−) ECs^a^ (only static significant) [67]
Long intergenic non-protein coding RNA 1094; ***LINC01094*** Long intergenic non-protein coding RNA 475**;** ***LINC00475/*** *C9orf44*	↑ TAVc vs. TAVn [182]
KLF3 antisense RNA 1**;** ***KLF3-AS1/*** *flj13197* *PGM5 antisense RNA 1;* ***PGM5-AS1*** */FAM233A (family with sequence similarity 233, Member A)* IL10RB antisense RNA 1 (head to head); ***IL10RB-AS1*** **/** *IFNAR2-AS1 (IFNAR2 Antisense RNA 1)/LOC100288432* Long intergenic non-protein coding RNA 1094; ***LINC01094*** */LOC100505702* Chromosome 8 Open Reading Frame 49; ***C8orf49/*** *G4DM (GATA4 downstream Membrane Protein)* *Mir-99a-let-7c cluster host gene;* ***MIR99AHG*** */ LINC00478/ C21orf34* ***FLJ38717*** */LOC401261- ncRNA*	↑ AS vs. control [114] ↑ AS vs. fibro(sclerotic) group [114]
APCDD1L Antisense RNA 1 (head to head); ***APCDD1L-AS1*** Long intergenic non-protein coding RNA 1013; ***LINC01013***	↑ TAVc vs. TAVn ↑ BAVc vs. TAVn [182]
MIR4435–2 host gene**;** ***MIR4435-1HG/*** *MIR4435–2HG*/*LOC541471*	↑ AS [99] ↑ TAVc vs. TAVn ↑ BAVc vs. TAVn [182]
Long intergenic non-protein coding RNA 1279; ***LINC01279*** Nuclear paraspeckle assembly transcript 1 (Non-Protein Coding); ***NEAT1/*** *LINC00084*	↑ BAVc vs. TAVn [182]
Cytoskeleton regulator RNA; ***CYTOR/*** *LINC00152/C2orf59/NCRNA00152*	↑ BAVc vs. TAVn [182] (NOTH1+/−) ECs^a^ (only shear significant) [67]
Metastasis associated lung adenocarcinoma transcript 1 (Non-Protein Coding); **MALAT** ***1/*** *LINC00047/NCRNA00047*	↑ AS [99] ↓ AS ↓ HAVICs [100]
TMEM161B antisense RNA 1; ***TMEM161B-AS1***/*AK082072 /linc-POLR3G-8* APOA1 antisense RNA; ***APOA1-AS1*** PTENP1 antisense RNA; ***PTENP1-AS*** SOX2 overlapping transcript; ***SOX2-OT/*** *DKFZp761J1324/NCRNA0004*	↓ Calcified TAV [46]
Long intergenic non-protein coding RNA 1896; ***LINC01896*** */LOC649504* Long intergenic non-protein coding RNA 1697; ***LINC01967*** */LOC284825* HLA complex group 11 (non-protein coding; ***HCG11***	↓AS [99]
Rhabdomyosarcoma 2 associated transcript (non-protein coding); ***RMST*** **/** LINC00054/*NCRMS*	↓ AS [99, 114]
HAND2 Antisense RNA 1 (Head to Head); ***HAND2-AS1/*** *NBLA00301*	↓ AS [99, 114] ↓ AS vs. fibro(sclerotic) group [114]
Prader Willi/Angelman region RNA 6; ***PWAR6/*** *LOC100506965*	↓ AS vs. fibro(sclerotic) group [114]
TRHDE antisense RNA 1; ***TRHDE-AS1***	↓ TAVc vs. TAVn ↓ BAVc vs. TAVn [182]
Long Intergenic Non-Protein Coding RNA 92; ***LINC00092*** **/** *NCRNA00092*	↓ TAVc vs. TAVn ↓ BAVc vs. TAVn [182] ↑ HAVICs^1^ [78]
Long intergenic non-protein coding RNA 894; ***LINC00894*** Long intergenic non-protein coding RNA 1354; ***LINC01354***	↓ BAVc vs. TAV [182]
OIP5 antisense RNA 1; ***OIP5-AS1***/Cyrano/LOC729082	↓ Calcified TAV [46] ↓ HAVICs^1^ [78]
Maternally Expressed 3 (Non-Protein Coding); ***MEG3*** **/** *LINC00023*	↓AS [114] ↓ HAVECs^c^ (FO/VL) [77]
HLA Complex P5 (Non-Protein Coding); ***HCP5***	↑ HAVECs^c^ (FO/VL; FO/FL) [77]
MIR503 host gene; ***MIR503HG/*** *H19X (H19 X-Linked Co-Expressed LncRNA)/ MIR503 Host Gene/ MGC16121* EPB41L4A Antisense RNA 1; ***EPB41L4A-AS1*** **/** *NCRNA00219/C5orf26/TIGA1*	↑ HAVECs^c^ (FO/VL; FO/FL; VO/VL) [77]
Long intergenic non-protein coding RNA 467; ***LINC00467/*** *C1orf97*	↑ HAVECs^c^ (FO/VL; FO/FL; VO/VL) [77] ↓ HAVICs^1^ [78]
ASTN2 antisense RNA 1; ***ASTN2-AS1/*** *LOC100128505* ***LOC100133669*** *-ncRNA* ***LOC729970*** */hCG2028352-like/ncRNA* IGFBP7 antisense RNA 1*;* ***IGFBP7-AS1/*** *LOC255130* Long intergenic non-protein coding RNA 294; ***LINC00294/*** *LOC283267* cancer susceptibility candidate 15 (non-protein coding); ***CASC15/*** *LINC00340/FLJ22536* Long intergenic non-protein coding RNA 2035; ***LINC02035/*** *LOC100129550*	↑ HAVICs^1^ [78]
HHIP antisense RNA 1; ***HHIP-AS1/*** *LOC646576* FAM13A antisense RNA 1; ***FAM13A-AS1/*** *NCRNA00039* KDM4A antisense RNA 1; ***KDM4-AS1/*** *LOC100132774* Long intergenic non-protein coding RNA 938; ***LINC00938/*** *LOC400027* Long intergenic non-protein coding RNA 998; ***LINC00998*** */LOC401397* Long intergenic non-protein coding RNA 623; ***LINC00623/*** *LOC728855* Long intergenic non-protein coding RNA 273; ***LINC00273/*** *LOC649159*	↓ HAVICs^1^ [78]
Neighbor of BRCA1 Gene 2 (Non-Protein Coding); ***NBR2/*** *NCRNA00192*	↑ PAVECs* [177]
Long intergenic non-protein coding RNA 862; ***LINC00862/*** *C1orf98*	HAVECs^b^ [63]

Legend: AS- aortic valve stenosis; BAVC- calcified stenotic bicuspid aortic valve; BAVr- bicuspid aortic valve with redundant leaflets and/or minimal calcification; HAVECs- human aortic valve endothelial cells; HAVICs- human aortic valve interstitial cells; FO-fibrosa, oscillatory shear stress; FL- fibrosa, laminar shear stress; (NOTH1+/−) ECs- hiPSC (human induced pluripotent stem cell)-derived endothelial cells generated from two patients with nonsense mutations in NOTCH1; PAVECs- porcine aortic valve interstitial cells; TAVc- calcified stenotic tricuspid aortic valve; TAVn -noncalcified tricuspid aortic valve, without stenosis; VL- ventricularis, laminar shear stress; VO- ventricularis, oscillatory shear stress. [46]- 9 tricuspid AS and 10 control nonmineralized aortic valves, male subjects; [184]- 5 tricuspid AS and 5 control nonmineralized aortic valves, male subjects; [92]- 10 BAVc, 9 TAVc and 8 control TAVn, male subjects; [101]- AS (5 TAVc/1BAVc), fibro(sclerotic) group (5 TAV/2 BAV), control (5 TAVn/1 BAVn), male subjects

^1^Genes altered > 1.2-fold with a *p* < 0.05 in the HOTAIR siRNA microarray data; ^a^NOTCH1 haploinsufficiency, shear or static flow significant; ^b^affected by flow and decreased NOTCH1; ^c^shear-sensitive transcripts; *shear stress conditions modeling laminar flow. NOTE: Official gene symbols are presented in bold and italicized, aliases are in italics

Implication of lncRNAs in AS development is also demonstrated by the role of intergenic lncRNA MALAT1 (metastasis-associated lung adenocarcinoma transcript 1) which is associated with osteogenic transdifferentiation of human VICs. Its upregulated expression in AS compared to control valves was first reported by Bosse et al. [99]. Contrary to them, Zhu et al. detected significantly lower levels of MALAT1, both in calcific valves and osteoblastic differentiating VICs accompanied by lower levels of TWIST1 (twist-related protein 1) protein [100]. Furthermore, they showed that MALAT1 directly interacts with TWIST1 and could enhance its stability while the TWIST1 overexpression prevents VICs calcification induced by inhibition of *MALAT1* [100]. Possible mechanism was previously indicated by study of Zhang et al. demonstrating that TWIST1 negatively regulates osteoblastic transdifferentiation of human VICs through direct inhibition of *RUNX2* [101].

#### LncRNAs associated with AS-induced LVH

Recent studies had shown that lncRNAs may also play an important role in regulation of AS-induced cardiac hypertrophy. Thus, Viereck et al. reported a significant upregulation of *CHAST* (cardiac hypertrophy-associated lncRNA transcript) lncRNA in hypertrophic heart tissue from AS patients and in human embryonic stem cell-derived cardiomyocytes upon hypertrophic stimuli [102]. Furthermore, Ounzain et al. reported the upregulation of *CARMEN* (cardiac mesoderm enhancer-associated noncoding RNA) in patients with idiopathic dilated cardiomyopathy (DCM) and AS [103]. The same group quite recently reported *WISPER* (WISP2 super-enhancer associated RNA) lncRNA as a novel cardiac fibroblast enriched super-enhancer related lncRNA, whose expression was significantly correlated with cardiac fibrosis both in a murine model of myocardial infarction and in AS patients [104]. Furthermore, previous study by Ounzain et al. reported differential expression of Novlnc6, Novlnc23, and Novlnc44 lncRNA in LV biopsy samples from dilated cardiomyopathy (DCM) and AS patients when compared to control LV tissue [105]. In contrast to DCM group, patients with AS were not associated with downregulation of enhancer derived lncRNA *Novlnc6*, or its predicted target gene *NKX2–5* (NK2 Transcription Factor Related, Locus 5). However, they detected significant downregulation of Novlnc44 while the downregulation of Novlnc23 was not statistically significant [105].

Contrary to the above, Peters et al. indicate no role of MALAT1 in PO-induced CH, myocardial remodeling and HF in mouse model(TAC- transverse aortic constriction, and angiotensin II-induced CH) of AS despite its reported role in regulation (binding and inhibition) of antihypertrophic miR-133 [106].

Other lncRNAs transcripts differentially regulated in AS compared to control aortic valve tissue are presented in Table 2. They are selected based on bioinformatic reanalysis of published data using *DAVID* database for annotation visualization and integrated discovery; *NCBI Gene* website; *Genecard*, the human gene database; *lncrnadb*, the reference database for functional long noncoding RNAs, and *HGNC web base* for gene nomenclature [88, 89, 107–110]. However, functional roles for most of these lncRNA transcripts in the vasculature and other human tissues are largely unknown.

#### MicroRNAs

MicroRNA (miRNA/miR) are the best studied epigenetic regulatory mechanism in AS and other cardiovascular diseases [26, 111–113]. Up to now, several qRT-PCR and miRNome-wide microarray studies, (Table 3) reported differential expression of more than 200 microRNAs in stenotic aortic valve leaflets or experimentally modified aortic valve cells [77, 87, 99, 114–124].

**Table 3:** Dysregulated microRNA in stenotic valves and experimentally modified aortic valve cells

MicroRNA expression	Method	Patients [reference]
Upregulated: miR-155/BIC/miR155HG, −21	Microarray	5 AS (TAV), 5 TAVn, male subjects [99]
Downregulated: **miR-16**, **−26a**, −27a, **−30b**, −130a, **−195**, **−497**	Microarray qRT-PCR	4 AS (BAV). 5 AI (BAV), male subjects [115]
Upregulated (ANOVA) TAVc vs. TAVn: miR-32*, 127-3e, −145, −483-5p, −572, −574-5p, −663, −671-5p, −1207-5p, −1224-5p, −1469, **−2861** ^**#**^, −3149, −3621 Upregulated (ANOVA) BAVc + R vs. TAVn: miR-466, −572, −671-5p, 1224-5p, −1207-5p, −1469, **−2861**, −483-5p, −127-3e, −4267, −663, −145, −3621, Upregulated (ANOVA) TAVn: let-7e, let-7i, −16, 29a^#^, **miR-29c,** **−30b** ^**#**^, **−30d** ^**#**^, −99a, −126, −100, −103, −107, −125b, −151-5p, −451, Overexpressed in disease (Mann Whitney U analysis): miR-143, −149*, −198, −483-5p, −572, −602, −638, −663, −671-5p, −762, −1207-5p, −1224-5p, −1231, −1275, −1307, −1469, −2861, −3665, −4270 Overexpressed in control (Mann Whitney U analysis): Let-7f, let-7 g, let-7i, miR-16, −25, −26b, −29a#, −29c, −30b, −30c, −30d, −30e, −99a, −100, −101, −106b, −107, −126, −140-5p, −199a-3p, −451, −486-5p, −4324	Microarray qRT-PCR	3 AS (TAV; 2 male), 5 BAVc +R (3 male), 4 TAVn (DCM patients, 2 males) [116]
Upregulated: miR-30e, −32, −145, −151-3p, −152, −190, −373*, −768-5p Downregulated: miR-22, −27a, −124-3pre, −125b-1-pre, **−141** (PAVICs), −185-pre-, −187, −194*, −211-pre, −330-5p, −370, −486-3p, −449b*, −551a-pre, −564, −566-pre, −575, −585-pre, −622, −637, −648-pre, −1202, −1282, −1469, 1908–1909*, −1972	Microarray qRT-PCR	19 BAVc (10 female) vs. 17 TAVc (11 female) PAVICs harvested from 10 porcine hearts [117]
Downregulated: miR-30a, −30b (HAVICs), −30c, −30d, −30e	qRT-PCR	10 AS (5/5 male/female, calcific vs. adjacent tissue) Primary HAVICs from 10 noncalcified human aortic valves [118]
Upregulated (cyclic stretched HAVICs): miR-132, −146a, −212, −486-5p, −941 Downregulated (BAV vs. control and cyclic stretch HAVICs): miR**-148a-3p -19b** Downregulated (cyclic stretch HAVICs): miR-19a, −143, −145, −148a-3p, −335*-, −374a*-, −450b-5p, −1197, −1280	MicroRNA-sequencing qRT-PCR	9 BAVc vs. 5 healthy aortic valves, male subjects 6 cyclic stretch stretched and 6 static HAAVIC samples [87, 119]
Upregulated: miR-21, −34b, −125a-5p, **−125b** ^*^ **, −193b,** −423-3p, −625* Downregulated: miR-124*, −184, −185*, −193a-5p, −374b*, −516a-5p, −519e*, −520d-5p, **−602** ^*^ **,** −637, **−665,** −921, **−939** ^*^	Microarray qRT-PCR	Microarray- 5 AS (1 BAV) vs. 5 control valves (2 BAVs), male subjects qRT-PCR- 20 AS vs. 6 control [114]
Upregulated: miR-29b-1-5p, −99b-3p, −193a-3p, −194-5p, −200b-3p, −505-5p Downregulated: miR-21-3p, −21-5p, −34a-3p, −146b-5p, −301a-3p, −3663-3p, −513a-5p, −516a-5p, −575, −630, −636, −718, −1972, −3138	Microarray	4 AS vs. 4 control, male subjects [120]
Upregulated: let-7f-5p, let-7i-5p, miR**-21-5p,** −27a-5p, −27b-3p, −31-5p, −34a-5p, −143-3p, −146b-5p, −199a-5p, −199a-3p, −199b-3p, −216a-5p, **−221-3p**, −222-3p, −335-5p, −381-3p, −455-3p, −548a-3p, −550a-3p, −1263, −1275, −3124-5p, −3128, −3178, −3197 Downregulated: miR-1, −16-5p, −17-5p, −18a-5p, −18b-5p, -19b-3p, −20a-5p, −20b-5p, −30d-5p, **−30e-5p,** −92a-3p, −93-5p, −99a-5p, −103a-3p, −106a-5p, −106b-3p, −107, **−122–5p**, −124-3p, −125b-2-3p, −128-3p, −133a-3p, −133b, −139-5p, −140-3p, −149-5p, −181a-2-3p, −182–5p, −185-5p, −191-5p, −192–5p, −194-5p, -197-3p, **−200c-3p** (↑qRT-PCR), −206, −214-3p, −320a, −320b, −320c, −324-3p, −328-3p, −339-3p, −339-5p, −378a-3p, −378a-5p, −378c, −422a, −425-5p, −451a, −486-3p, **−486-5p** (no difference in qRT-PCR), −491-5p, −500a-3p, −500a-5p, −501-3p, −502-3p, −532–5p, −532-3p, −574-3p, **−625-5p**, −629-5p, −652-3p, −664-5p, −665, −766-3p, −885-5p, −933, −939-5p, -1180-3p, −1202, −1207-5p, −1225-5p, −1246, −1271-5p, −1287-5p, −2110, −1973, −3162–5p, −4253, −4284	Microarray qRT-PCR	Microarray- 15 AS (9male) vs. 16 control (12 male) qRT-PCR 36 (25 male) vs. 16 (11 male) [121]
SAM: Upregulated: (FO/VL): miR**-187** ^**$**^, **−217** ^**T**^ **, −374a**; (VO/VL): −187, −217; (FO/FL): −187, −769-3p; SAM Downregulated (FO/VL): miR**-139-3p** ^**$**^, **−382**, **−433, −483-3p**, **−485-3p**, **−485-5p**, **−486-3p**, −**486-5p** ^**$**^ (Up qRT-PCR), **miR**-**543**, **−549** (Up qRT-PCR), **miR-923**, **−1237** (Up qRT-PCR), **miR**-**1244**; (VO/VL): miR-139-3p, **−192** ^**$**^, −411, −486-3p, −486-5p, −518e:9.1, −548o, −647, −654-3p, −923, −1244, −1290, −1300; (FO/FL): miR-486-3p, −486-5p, −923, −1244; (FO/VO): **miR-485-3p** (side dependent), **miR-485-5p** (side dependent); (FL/VL): **miR-370** ^**$**^ (side dependent) Additional analysis- Downregulated (VO/VL): **miR-192** (previously found as a shear-sensitive miRNA) Additional 15 miRNAs, chosen based on fold change and importance in other data sets: Upregulated (FO/VL; qRT-PCR): **miR-15a**, **−29c** (down qRT-PCR), **miR-106b**, **−126**, **−148a** ^**$**^, **−186**, **−365**, **−424** ^T^, **−495** (down qRT-PCR); Downregulated (FO/VL; qRT-PCR): **miR-10a, −214, −615-5p** (unchanged in qRT-PCR), **miR-663b** (Up qRT-PCR), **miR-92b** (Up qRT-PCR); Unchanged**: miR-92a** (Up qRT-PCR)	Microarray qRT-PCR	HAVECs from healthy noncalcified valves [87, 122]
SAM: (13 human shear-responsive and side specific out of 24 unique side-specific) miRNAs: **miR-100 (**Up F/V qRT-PCR, miRNA array), miR-150, **−181a (**Up F/V qRT-PCR, miRNA array), **miR-181b (**Up F/V qRT-PCR, miRNA array), miR-**199a-3p (**Up F/V qRT-PCR, miRNA array), **miR-199a-5p (**Up F/V qRT-PCR, miRNA array), miR-199b-3p, **−214 (**Up F/V qRT-PCR, miRNA array), miR-223, −455-3p, −523-star, −618, −708 Additional analysis: Upregulated F/V: **miR-130a**; **miR-192** ^**T**^, **−486-5p** ^*^	Microarray qRT-PCR FISH	PAVECs from healthy noncalcified valves [122, 123]
FISH in situ hybridization in cryosections of porcine aortic valve: miR-486-5p- trend for increased staining on the fibrosa side Upregulated F vs. V at total (mature and pre-miR forms) miRNA level: **miR-100** ^**$**^, −**130a** ^**$**^ (homologous to human miR-130a-3p), −**181a** ^**$**^ (homologous to human miR-181a-5p)**, −181b** ^**$**^ (homologous to human miR-181b-3p), −**199a-3p** ^**$**^, −**199a-5p** ^**$**^, **−214** ^**$**^ (homologous to human miR-214-3p) Upregulated F vs. V at the mature miRNA level: **miR-181a** ^**T**^ **, −199a-5p** ^*^ **, −199a-3p** ^**T**^ **, −214** ^**$**^ Upregulated FO/FL^*^, VO/VL, FO/VL^*^, FO/VO^*^, FO/fresh valve^*^: **mir-214** (side- and shear-dependent)	Microarray qRT-PCR	

Legend: AS- aortic valve stenosis; AI- aortic insufficiency; BAVn- healthy noncalcified bicuspid aortic valve; BAVC- stenotic calcified bicuspid aortic valve; BAVc + R- stenotic bicuspid valves in which a raphe was visible; BIC- B-cell receptor inducible, miR155 host gene; DCM dilated cardiomyopathy; HAVECs- human aortic valve cells; HAVICs- human aortic valve interstitial cells; PAVECs- porcine aortic valve cells; FO-fibrosa, oscillatory shear stress; FL- fibrosa, laminar shear stress; FISH- fluorescent *in situ* hybridization; qRT-PCR- quantitative real time polymerase chain reaction; SAM-Significance of Microarray Analysis; TAVc- calcified stenotic tricuspid aortic valve; TAVn -noncalcified tricuspid aortic valve; VL- ventricularis, laminar shear stress; VO- ventricularis, oscillatory shear stress; miRs- higher expression in TAVc vs. BAVc; **miRs designated in bold﻿-** miRs evaluated by qRT-PCR. ^$^statistically significant, qPCR of additional miRNAs yielded miR-148a as shear-sensitive (not found in SAM); ^T^trend toward significance; ^#^miRNA with similar expression profile in both diseased groups when compared to healthy aortic valves

Numerous functional studies (Table 4 **)** revealed that many of these miRNA transcripts are involved in activation and osteogenic differentiation of AVICs [92, 115–138]. In addition, these ossification-related miRNAs, so called osteomiRs, have also a prominent role in calcification of other cardiovascular tissue settings [139–146].

**Table 4:** Functional analysis of dysregulated microRNAs in stenotic aortic valves and experimentally modified aortic valve cells

Dysregulated microRNA	Source	Role	Reference
↓ miR-19b	BAVc, HAVICs (cyclic stretch)	MiR-19b mimic (HAVICs) → modulation of osteogenic TGFβ signaling: ↓ TGFBR2, IGF1 (HAVICs under cyclic stretch), relative ↑ SMAD3^*^/ SMAD5^*^, ↑ ALP^*^ mRNA	[119]
↓ miR-26a	BAVC, diseased and healthy HAVICs	MiR-26a mimic (HAVICs) → pro-calcification related genes: ↓ALP*, ↓BMP2*, ↓SMAD1*, ↓BMP4^T^; ↑RUNX2* ↑SMAD5*; anti-calcification related genes ↑JAG2*↑SMAD7*	[118]
↓miR-29a/c	BAVc, BAVc +R, TAVc	↓miR-29a/c (BAVc, BAVc +R, TAVc) → ↑Collagen 1, ↑Collagen 3	[116]
↓ miR-30b	BAVc, diseased and healthy HAVICs	MiR-30b mimic (HAVICs) → pro-calcification related genes: ↓SMAD1*, ↓SMAD3*; anti-calcification related genes: ↑JAG2*, ↑SMAD7*, ↓NOTCH1*	[115]
	Calcific AS valves	MiR-30b mimic (HAVICs) → reduce BMP2-induced osteoblast differentiation: ↓ RUNX2, ↓ SMAD1, ↓ CASP3; ↓ ALP activity, ↓BGLAP/OCN	[118]
	BAVc, BAVc +R, TAVc	↓miR-30b(c/d) (BAVc, BAVc +R, TAVc) → ↑ RUNX2	[116]
miR-30e	Aortic valves	Injections of antimiR-30e in ApoE−/− mice → ↑ IGF2 (aorta, liver), ↑ OPN^*^ protein expression and ↑ calcium deposition^*^ in aortic valves	[125]
↑ miR-125b	TAVc/BAVc (5/1), cultured human THP1 macrophages	miRNA-125b transfection (human THP1 macrophages) → ↓ CCL4^*^	[92]
↓ miR-141	BAVc, TAVc, PAVICs	↓ miRNA-141 (BAVc vs. TAVc) miRNA-141 transfection (PAVECs) → ↓ BMP2, represses TGFβ–triggered PAVIC response to injury and calcification	[117]
↑ miR-143	Human and murine model of AVSc	↑ miR-143 (VICs exposed to oxidative damage in the presence of SOD mimetics and AV explants) With SOD mimetics mediates the pathological valve remodeling (matricellular protein expression αSMA, OPN) in a murine model of AVSc	[126]
	Osteogenic-induced (TGβ1) VICs	C57BL/6 J mice injected with LNA-miR143 after the development of AV thickening (after 4–8 weeks of ANG II infusion that mimic AV remodeling a in AVSc) have reduced AV peak gradient, peak velocity, and velocity-time-interval. in silico target prediction revels miR143 as a regulator of OPN-CD44 axis that mediates calcium deposition via phospho-AKT in HAVICs from patients with noncalcified AVSc	[127]
↓ miR-148a-3p	BAVc, cyclic stretch HAVICs	Cyclic stretch (HAVICs) → ↓ miR-148a-3p → ↑ NF-κB → activates NF-κB dependent inflammatory signaling pathways MiR-148a mimic transfection (HAVICs) → ↓ IKBKB; ↓NF-κB signaling, ↓NF-κB target gene expression → ↓ IL1B, ↓ IL8, ↓ MMP1, ↓MMP14, ↓ MMP16	[119]
↑ miR-181a	Porcine AV leaflets (cyclic stretch vs. static conditions)	↑ miR-181a (15% cyclic stretch porcine AV leaflets) → ↓ ALP^*^, ↓ BGLAP/OCN^*^	[128]
↑ miRNA-181b	Aortic valve endothelium	Shear-sensitive miRNA-181b impairs anti-inflammatory signaling in the aortic valve endothelium ↑ miRNA-181b (AVECs in OS conditions) correlates with: ↑ inflammatory adhesion molecules, ↓anti-inflammatory marker KLF2 OS → ↓ predicted miRNA-181b target OGT → decreased binding of OGT to MEF2C → inhibition of MEF2C O-GlcNAc modification	[129]
miR-187	HAVECs	Overexpressed miR-187 in vHAVECs → significant decrease in monocyte adhesion in vHAVECs exposed to LS → reduction in inflammatory state	[122]
↓ miR-195	BAVc, diseased and healthy HAVICs	MiR-195 mimic transfection (HAVICs) → pro-calcification related genes: ↑BMP2*, ↑RUNX2*; ↑SMAD1*, ↑SMAD3*, ↑SMAD5*; anti-calcification related genes: ↑JAG2*, ↑SMAD7*	[115]
↓ miR-204	AS and HAVICs	↓ miR-204 (AS and BMP2 treated HAVICs) → ↓ RUNX2 miR-204 mimic transfection (BMP2 treated HAVICs) → ↓ ALP^*^ ↓ BGLAP/OCN^*^, ↓ BMP2 induced RUNX2 mRNA and protein levels	[130]
	Healthy and diseased HAVICs	TGFβ1 and BMP-2 treated HAVICs → ↓ miR-204 → ↑ RUNX2, ↑ SP7/OSX Mir-204 mimic → ↓ RUNX2, ↓ SP7/OSX	[131, 132]
↑ miR-214	Porcine AV leaflets (cyclic stretch vs. static conditions)	↑ miR-214 (15% cyclic stretch porcine AV leaflets) → ↓ ALP^*^, ↓ BGLAP/OCN^*^	[128]
	PAVECs	anti-miR-214 (whole AV leaflets with the fibrosa exposed to OS) → ↑ TGFβ1*, moderate ↑ collagen content, not effect on AV calcification	[123]
	AS and HAVICs Hypercholesterolemic ApoE−/− murine AS model M1/M2 macrophage	↑ miR-214 accompanied with valve calcification and M1 macrophage polarization M1 macrophage-derived microvesicles deliver miR-214 to HAVICs → pro-osteogenic differentiation, ↓ TWIST1 → aortic valve calcification intravenous treatment of hypercholesterolemic male ApoE−/− mice with a miR-214 inhibitor → significant suppression of valve calcification, ↑ TWIST1	[134]
miR-483-3p	HAVECs	↓miR-483-3p (HAVECS subjected to OS) → ↑ ASH2L ↑ miR-483-3p (HAVECS subjected to LS) → ↓ ASH2L	[135]
↑ miRNA-486	TGF-β1 and BMP-2 treated HAVICs	TGFβ1 and BMP-2 treated HAVICs → ↑ miR-486 miR-486 mimic (TGFβ1 and BMP-2 treated HAVICs) → ↑ RUNX2, ↑ SP7/OSX	[131, 132]
	Healthy and diseased HAVICs	miR-486 mimic (HAVICs) → ↑α-SMA through modulation of PTEN-AKT pathway, ↑ MYLK →cell aggregation, fibroblast-to-myofibroblast HAVICs transition and calcification nodule formation Prolonged miR-486 treatment (healthy HAVICs) → ↑ collagen I, ↑ MMP2 and ↑ MMP9.	[132, 133]
↑ miR-486-5p	HAVECs Porcine ventricularis	↑ miR-486-5p (HAVECs subjected to LS, porcine ventricularis) → ↑ cell migration, ↓ apoptosis Potential targets: EFNA1 and PRND – role in endothelial-to-mesenchymal transition and oxidative stress	[136]
miR-1237-3p	Healthy HAVECs porcine aortic valves	differential expression between OS (↓ miR-1237-3p) and LS (↑ miR-1237-3p) miRNA1237-3p mimic → ↓ monocyte binding ↓VCAM1, ↓IL1β in static HAVECs	[137]
		↑ miR-1237-3p (HAVECs subjected to LS) → ↓CXCL2, ↓CXCL12, ↓NOX4, ↓ THBS1 → ↓ inflammation, endothelial dysfunction, valve calcification ↓ miR-1237-3p (HAVECs subjected to OSS) → ↑CXCL2, ↑CXCL12, ↑NOX4, ↑ THBS1 → ↑ inflammation, endothelial dysfunction, valve calcification	[138]
miR-2861	BAVc, BAVc +R, TAVc	↑ RUNX2, probably by targeting its inhibitor HDAC5	[116]

Legend: ALP- alkaline phosphatase; ANG II- angiopoietin 2; ApoE- apolipoprotein E; AS- aortic valve stenosis; ASH2l- ASH2 like histone lysine methyltransferase complex subunit; AV- aortic valve; AVSc- aortic valve sclerosis; BAVc- stenotic calcified bicuspid aortic valve, BAVc + R- stenotic calcified bicuspid valves in which a raphe was visible; BGLAP/OCN- osteocalcin; BMP2/4- bone morphogenetic protein 2/4; CASP3- caspase 3;CCL4- C-C motif chemokine ligand 4; CXCL2- C-X-C motif chemokine ligand 2; CXCL12- C-X-C motif chemokine ligand 2; EFNA1- Ephrin A1; HAVICs- human aortic valve interstitial cells; HDAC5- histone deacetylase 5; IGF1- insulin like growth factor 1; IGF2- insulin like growth factor 2; IKBKB- inhibitor of kappa light polypeptide gene enhancer in B-Cells, Kinase Beta; IL1β- interleukin 1 beta; IL8- interleukin 8; JAG2- Jagged 2; LNA-miR- locked nucleic acids resistant to exo- and endonucleases resulting in high stability in vivo and in vitro and increased target specificity; KLF2- Kruppel like factor 2; LS- laminar shear stress; NF-κB- nuclear factor kappa-light-chain-enhancer of activated B cells; NOX4- NADPH Oxidase 4; MMP2/9/14/16- matrix metalloproteinase 2/9/14/16; MYLK- myosin light chain kinase; OGT- O-linked N-acetylglucosamine; SPP1OPN- osteopontin; OS-oscillatory shear stress; PAVCs- porcine aortic valve; PRND- Prion Protein 2 (Dublet); RUNX2- Runt related transcription factor 2; SMAD1/3/5/7- SMAD Family Member 1/3/5/7; SOD- superoxide dismutase; SP7/OSX- osterix; TAVc- stenotic calcified tricuspid aortic valve; TGFBR2 –transforming growth factor beta receptor 2; THBS1- thrombospondin 1; TWIST1- Twist family BHLH transcription factor 1; VCAM1- vascular cell adhesion molecule 1; ^*^Statistically significant; ^T^ trend toward significance

For example, Balderman et al. have reported that BMP2 decreases microRNA-30b and microRNA-30c thus promoting calcification of VSMCs [140, 141]. In addition, Ding et al. reported that miR-30e represses the osteogenic program (reduction of the osteogenic panel: dermatopontin/*DPT*, decorin/*DCN*, *RUNX2*, *BMP4*, *SPP1/OPN*, *IGF2*/insulin-like growth factor 2, *ALP*, and *BGLAP/OCN*) in bone marrow-derived mesenchymal stem cells and SMCs by targeting *IGF2* and drives their differentiation into adipogenic or SMC lineage [125]. They showed that injections of antimiR-30e increases *IGF2* expression in the mouse aorta and significantly enhances *OPN* protein expression and calcium deposition in aortic valves [125]. They also showed that *NFYC* (nuclear transcription factor gamma subunit c) gene and its hosted miR-30e transcripts are down-regulated with age and atherosclerosis and inversely proportional to the expression of the osteogenic genes *RUNX2, OPN*, and *IGF2* [125]. MiR-30b/c/d transcripts were also predicted to target *RUNX2*, while miR-29a and miR-29c were known to target collagen production and miR-2861 has been shown to affect RUNX2 activity (inhibitor of RUNX2 protein) in mouse osteoblasts by targeting HDAC5 [116, 142]. In addition, Hu et al. and Xia et al. have detected the regulatory feed-back loop between RUNX2, miR-3960 and miR-2861 acting during the osteoblastic differentiation and in osteogenic transdifferentiation of VSMCs, correspondingly [143, 144]. Their results suggested that RUNX2 could directly induce the expression of miR-3960/miR-2861 cluster (located at the same loci) by binding to the putative binding site of its promoter [143, 144]. Furthermore, Goettsch et al. reported the involvement of miR-125b in osteogenic transdifferentiation of VSMCs in in vitro and in vivo experimental settings by targeting the osteoblast transcription factor *SP7/OSX* while When et al. showed that miR-125b regulates transdifferentiation and calcification of VSMCs in a high-phosphate environment by targeting *ETS1* (ETS proto-oncogene 1), a known transcription factor involved in osteoblastogenesis and extracellular matrix (ECM) mineralization [145, 146]. However, the applicability of these findings in the settings of AS largely remains to be established.

MiRNA transcripts dysregulated in AS have also multifactorial impact on endothelial dysfunction, inflammation, and endothelial-dependent myofibroblastic activation and osteoblastic transdifferentiation of AVICs (Table 4). For instance, Ohukainen et al. detected miR-125b as one of the most prominent dysregulated miRNAs in AS compared to control valve tissue. They also detected an increased expression of the inflammatory chemokines *CCL3* (chemokine (C-C motif) ligand 3) and *CCL4* (chemokine (C-C motif) ligand 4) in macrophages and αSMA-positive myofibroblasts and confirmed the *CCL4* as a direct target of miR-125b in cultured human THP-1 macrophages, thus showing the connection between microRNA and inflammatory gene expression in AS [92].

Various flow-sensitive miRNAs, known as mechano-miRs, are also detected in stenotic valves. For instance, Patel et al. have identified 5 upregulated and 10 down regulated miRNAs (Table 3) in human BAV tissue and AVICs exposed to cyclic stretch [87, 119]. They also found that stretch-modulated repression of miR-148a-3p and miR-19b (Tables 3 and 4) may be sufficient to activate macrophages and to promote inflammatory and osteogenic signaling pathways in AVICs [87, 119]. Shear and side-dependent miRNAs that regulate key mechanosensitive genes were also detected in human and porcine AVECs (Tables 3 and 4) [77, 122, 123].

Altered expression of endothelial mechano-miRNAs is also detected by Theodoris et al. in hiPSC-based modeling of human NOTCH1 mutations in AS [67]. They reported a significant dysregulation of 181 small ncRNAs in NOTCH1+/− compared to WT ECs in static versus shear stress conditions. Among them miR-30d, miR-663, miR-1260b and miR-3960 were upregulated while miR-20a/b, miR-21, miR-26a, mir-29c, miR-30e, miR-106a and miR-126 were downregulated in *NOTCH1+/−* ECs [67].

Moreover, differential expression of miRNAs was also detected in circulatory osteoprogenitor cells that play a significant role in pathogenesis of AS disease. Thus, a recent study reported by Takahashi et al. detected a higher levels of pro-osteogenic miR-30c in the AS group compared to controls and lower levels of miR-31, miR-106a, miR-148a, miR-204, miR-211, and miR-424, previously reported as negative regulators of pro-osteogenic pathways in mesenchymal stem cells [147–150]. Also, the degree of aortic valve calcification in their samples was weakly positively correlated with the number of COPCs and miR30c levels. Furthermore, the number of COPCs and the level of BGLAP/OCN protein in these cells was positively correlated with miR-30c and negatively correlated with the levels of remaining miRNAs [147]. Importantly, after surgical procedure both the number of COPCs and the levels of miR30c were decreased while the levels of the other miRNAs remained the same [147]. Moreover, the observed changes in miRNAs levels were greater after AVR than TAVR procedure. That may be explained by the procedural differences between these two surgical procedures, with TAVR leaving the residual diseased tissue around the prosthetic valve, which may continue to activate osteogenic processes via dysregulation of ossification related miRNAs [147].

Finally, Li et al. recently reported the role of macrophage miRNAs in regulation of AS [134]. They found that M1 macrophage-derived microvesicles deliver miR-214 promote pro-osteogenic differentiation of VICs and subsequent aortic valve calcification through downregulation of *TWIST1*, a direct target of miR-214 [134]. The upregulation of miR-214 of aortic valve samples was accompanied with both valve calcification and M1 macrophage polarization. In addition, the functional involvement of miR-214/*TWIST1* in the osteogenic differentiation of VICs was further confirmed by intravenous treatment of hypercholesterolemic APOE−/− mice with a miR-214 inhibitor, which significantly suppressed valve calcification and resulted in the upregulation of *TWIST1* [134].

#### Circulatory microRNAs

The utility of circulating miRNAs, as potential diagnostic, and prognostic biomarkers of AS, has also attracted considerable attention over the recent years. They have been correlated both with LV structural and functional impairment and with the outcome of AS patients after surgery.

For instance, the role of circulatory miR-21 as an indicator of LV fibrosis in AS patients in response to PO was supported by findings of Villar et al. and Fabiani et al. while Coffey et al. reported the higher levels of circulatory miR-21-5p in AS patients without coronary artery disease (CAD), but no apparent difference was found between groups with CAD [151–153]. Moreover, they showed that plasma levels of miR-21 and miR-21-5p correlated directly with the mean transvalvular gradients thus underscoring the value of circulating miR-21 as a biomarker for MF [151–153].

Coffey et al. detected higher circulatory levels of miR-451a and miR-22-3p, and lover levels of miR-24-3p and miR-382–5p in AS patients compared to controls that also remained significantly after adjusting for age [153]. However, after qRT-PCR validation only miR-22-3p and miR-382–5p had levels expected from microarray analysis. In addition, miR-22-3p and miR-24-3p were increased, whereas miR-382-3p was reduced in AS participants with CAD [153]. Furthermore, similar to miR-21-5p, the circulatory levels of miR-382–5p in AS patients also showed a significant correlation with maximum transvalvular velocity and mean gradient, but not with LV mass index [153].

Significantly lower circulating levels in AS patients compared to healthy controls were also reported for miR-1, miR-133, and miR-378 by Chen et al. [154]. In addition, their results indicated that the lower levels of miR-378 may serve as predictor for LVH independent of the pressure gradient [154]. Similarily, Garcia et al. reported that the higher preoperative plasma levels of miR-133a can predict the reversibility of LV hypertrophy after AVR, while Røsjø et al. detected positive association between higher circulating levels of miR-210 and increased mortality during follow-up of AS patients independently of established risk indices, including NT-proBNP (N-terminal proBrain Natriuremic Peptide) levels [155, 156].

Quite recently Martínez-Micaelo et al. reported that plasma circulating miRNA expression profile in AS patient may also reflect the morphology of the aortic valve (bicuspid/tricuspid) [124]. Their miRNA-wide microarray and qRT-PCR comparison between the groups (healthy TAV subjects without aortic dilation, BAV patients without aortic dilation and BAV patients with aortic dilation) revealed that the expression levels of circulating miR-122, miR-130a and miR-486 are significantly influenced by the morphology of the aortic valve (BAV vs. TAV), while the expression pattern of miR-718 was inversely correlated with the aortic diameter and thus may possibly be used an independent predictor of aortic dilation [124]. In addition, the targeted gene prediction and putative function analysis of selected miRNAs revealed that the bicuspid valve morphology-associated miRNAs (miR-122, miR-130a and miR-486) most probably affects the TGFβ1 signaling pathway (a total of 32 targeted genes) while the dilatation related miR-718 may be associated with focal adhesion and blood vessel remodeling processes [124].

Another, blood based microarray profiling reported by Derda et al. detected that among 8 known cardiovascular miRNAs (miR-1, miR-21, miR-29a, miR-29b, miR-29c, miR-133a, miR-155 and miR-499) only miR-29a, and miR-29c have potential to distinguish between patients with AS and hypertrophic non-obstructive (HNCM) and obstructive (HOCM) cardiomyopathies [157]. More specifically, they found increased levels of miR-29a in HOCM patients that correlated markers of cardiac hypertrophy while miR-29c was upregulated in AS but not in the other patient groups [157].

The possibility that circulating miRNAs are differentially expressed in the blood of hypertrophic cardiomyopathy patients and those with LVH induced by AS has been further investigated by Roncarati et al. [158]. Among the miRNAs significantly increased in the plasma of their patients (miR-27a, miR-199a-5p, miR-26a, miR-145, miR-133a, miR-143, miR-199a-3p, miR-126-3p, miR-29a, miR-155, miR-30a, and miR-21) they found that correlation with LVH holds true for only miR-199a-5p, miR-27a, and miR-29a, whereas only miR-29a was significantly associated with both hypertrophy and fibrosis, thus identifying it as a potential biomarker for myocardial remodeling assessment in CH [158]. The similar trend was detected for only five miRNAs (miR-21, miR-26a, miR-27a, miR-30a, and miR-133a) in AS patients, whereas mir-29a levels showed no increased in AS relative to control, thus suggesting specific miR signatures for these two pathological conditions [158].

Interestingly, Varrone et al. reported that plasma levels of mRNA protein targets in AS patients may also be used for indirect measurement of myocardial miRNAs expression. They detected inverse relationship between myocardial expressions of miR-1 and circulating levels of (FABP3, heart-type fatty acid-binding protein-3) in AS patients with LVH [159]. Specifically, myocardial miR-1 expression was decreased while FABP3 levels were increased compared to controls [159]. Furthermore, the decrease of myocardial wall stress following TAVR procedure have led to downregulation of FABP3 levels to values comparable to ones for control subjects [159]. Moreover, the increased level of circulating IGF1 (insulin-like growth factor 1) in AS patients were also significantly blunted by the TAVR procedure, thus suggesting that this reciprocal relationship between miR-1 and FABP3 protein may be tightly controlled by the IGF1/miR-1/FABP3 signaling axis [159]. It seems that the hypertrophic response in AS patients is followed by the increase in IGF1 plasma levels and downregulation of miR-1 [159]. Eventually, the reduction of cellular miR-1 levels relieves its negative control over the expression of *FABP3*, thus leading to a prompt protein release into the circulation where it might be used for indirect measurement of myocardial miR-1 activity [159].

#### Pericardial fluid miRNAs

Several vascular-enriched miRNAs have also been detected in pericardial fluid (PF) of AS patients undergoing surgery. For instance, Miyamoto et al. reported significantly higher levels of miR-423-5p in PF compared to its plasma values [160]. Contrary, the levels of muscle-enriched (miR-133a) and vascular-enriched (miR-126 and miR-92a) miRNAs were found unaltered [160].

In another experiment, Kuosmanen et al. detected more than 70 miRNAs in PF collected from AS and other HF patients (coronary artery disease, mitral valve insufficiency, aortic valve insufficiency, and other cardiovascular disease) during open-heart surgery, with let-7b-5p, miR-16-5p, miR-21-5p, miR-125b-5p, and miR-451a being the most abundant [161]. However, despite the differences in disease etiologies (ischemic vs. nonischemic) or the stage of the HF the overall miRNA profiles were quite similar between the groups [161].

The existence of functional miRNAs in PF samples of AS patients was recently reported by Beltrami et al. [162]. Among the 359 detected miRNAs in PF and patient matched peripheral plasma samples they confirmed PF exosome enrichment for 15 of them (let-7b-5p, miR-15a-5p, miR-16-5p, miR-19b-3p, miR-21-5p, miR-21-5p, miR-23a-3p, miR-24-3p, miR-27a-3p, miR-27b-3p, miR-29a-3p, miR-29b-3p, miR-29c-3p, miR-126-3p, miR-451a) [162]. These miRNAs were also found co-expressed in patient’s myocardium and ascending aorta [162]. Furthermore, at functional level these PF derived exosomes were able to improve survival, proliferation, and networking of cultured endothelial cells, restore their angiogenic capacity and promote post-ischemic blood flow recovery and angiogenesis in mice models, all of which was partially mediated by exosomal transfer of let-7b-5p miRNA and decreased transcription of its targeted gene *TGFBR1* [162]. Thus, it seems that PF enriched cardiovascular miRNAs may act as endocrine and paracrine signaling factors responsible for local crosstalk between cardiac cells, and some of them may be utilized to reflect the patient clinical status.

#### Myocardial miRNAs

Expressional profiling of myocardial biopsies from AS patients further confirms the regulatory role of miRNAs in development of LVH and fibrosis induced by AS [163].

The role of myocardial miR-21 in these processes was supported by several investigators. Villar et al. reported that AS patients featured higher myocardial expression levels of both primary (pri-miR-21) and mature miR-21 transcripts. They were restricted to the interstitial cells (cardiac fibroblasts) within the ECM, with no or very weak miR-21 signals detected in the cardiomyocytes of AS patients or control samples, correspondingly [151]. Moreover, both the myocardial and circulating levels of miR-21 were positively correlated with the myocardial expression levels of genes encoding collagen I, collagen III, fibronectin, *TGFβ1* and effectors of TGFß signaling (*SMAD2* and *MAP3K7/TAK1* mitogen-activated protein kinase kinase kinase 7) together with negative correlation with the miR-21 targets (*PTEN*, phosphatase and tensin homolog; *TIMP3*, tissue Inhibitor of metalloproteinases; *RECK*, reversion inducing cysteine rich protein with kazal motifs, and *PDCD4* programmed cell death 4/neoplastic transformation inhibitor), thus suggesting a link between the severity of the maladaptive remodeling and miR-21 upregulation in the myocardium [151]. Another study reported by Lorenzen et al. detected the link between increased *OPN* expression and activation of the transcription factor *AP-1*, with subsequent miR-21 induction and regulation of downstream antifibrotic targets (*PTEN* and *SMAD7*) in angiotensin 2 (ANGII/AGT)-induced cardiac cells and in LV biopsies from AS patients with myocardial fibrosis [164]. In addition, Garcia et al. reported a new TGFβ-dependent regulatory mechanism involved in the miR-21 transcription and posttranscriptional maturation, through the interaction of p-SMAD2/3 effectors with the ribonuclease DICER1 processor machinery [165]. They showed that this TGFβ-dependent facilitator mechanism could contribute to the pathogenesis of PO-induced myocardial remodeling both in the experimental mouse model with transverse aortic constriction (TAC), and in patients with AS [165].

The association of miRNAs with PO-induced cardiac hypertrophy and heart failure in mouse model (TAC) of AS has also been reported by Eskildsen et al. [166]. Together with several well-described cardiac disease related miRNAs (miR-21, miR-29, miR-133a, and miR-208b), they identified altered expression of three novel miRNAs; miR-24, miR-301a, and miR-335 in the left ventricle of TAC affected mice compared to controls [166]. Interestingly, the increased expression of miR-24, miR-301a, and miR-335 was not found in an animal model of myocardial infarction thus suggesting that their regulation is specific for AS and is not part of a general cardiac disease response [166]. Furthermore, the importance of miR-133a in regulation of AS induced LVH was also reported by Duisters et al. [167]. They found reduced miR-133 and miR-30c expression levels in several forms of PO–induced LVH including the hearts of AS patients [167]. In addition, the expression of both miRNAs was inversely related to the protein level of CTGF (connective tissue growth factor), a key molecule in the process of fibrosis and a powerful inducer of extracellular matrix (ECM) synthesis [167]. Moreover, several lines of evidence provided by this study confirmed a negative regulatory role of miR-133 and miR-30c on the levels of *CTGF* gene expression, not only by repressing *CTGF* translation but also by degrading its mRNA [167]. Interestingly, 34 of the 42 mammalian collagen genes are also predicted targets of miR-133, thus suggesting a major role for this miRNA in preventing cardiomyocyte collagen synthesis, and the quality of their surrounding ECM [167].

Recently, another study reported by Jiang et al. detected the negative correlation between miR-133 and lncRNA-ROR (regulator of reprogramming, also named lncRNA-ST8SIA3) in mouse (TAC) model of pressure overload induced cardiac hypertrophy [168]. Thus, it seems that lncRNA-ROR serves as the miR-133 sponge while on the other hand overexpression of miR-133 successfully attenuates the expression of lncRNA-ROR reversing its pro-hypertrophic influence leading to markedly decreased expression of fetal *NPPA/ANP* (natriuretic peptide A) and *NPPB/BNP* (natriuretic peptide B) genes [168]. In addition, Renaud et al. reported that members of class I and IIb HDACs may also play a role in the regulation of PO-induced miR-133a downregulation in mouse (TAC) model of cardiac hypertrophy [169]. More specifically, the treatment with the class I and IIb HDAC inhibitor Vorinostat (also known as SAHA- suberoylanilide hydroxamic acid) significantly attenuated TAC-Induced downregulation of miR-133a and diminished the upregulation of CTGF protein abundance and collagen deposition, thus suggesting that the effect of HDAC inhibition on miR-133a expression is reflected on its downstream fibrotic targets [169].

These two reports clearly indicate the importance of understanding the complex crosstalk between miRs and other epigenetic regulators such as lncRNAs and HDACs, thus providing the ground for innovative therapies to reset the epigenome alterations in AS and other heart diseases.

MiR-133a also emerged as a key element of the postoperative reverse remodeling process of LVH in the report of Villar et al. [170]. They identified the microRNA-133a levels in intraoperative biopsies as a significant positive predictor of left ventricular mass normalization in AS patients while β-myosin heavy chain expression and BMI constituted negative predictors [170].

Furthermore, Beaumont et al. have reported that myocardial down-regulation of miR-122 might also be involved in the development of myocardial fibrosis in AS patients, most probably through the up-regulation of TGFβ1 [171]. They also reported differential expression of 118 miRNAs (99 down-regulated and 18 up-regulated) in AS patients with severe myocardial fibrosis (SF) compared with the non-SF AS group [171]. The role of miRs in regulation of TGFβ pathway in the cardiomyocyte hypertrophy and interstitial fibrosis in the settings of AS was also reported by Tijsen et al. [172]. They showed that the members of the miR-15 family (miR-15a/b, miR-16, miR-195, miR-497, and miR-322), that are abundantly expressed in the heart and up-regulated in the diseased myocardium, directly or indirectly inhibit TGFβ-pathway by targeting *TGFBR1* and several other genes (*p38*, *SMAD2*, *SMAD3*, *SMAD7*, and Endoglin/*ENG*) within this pathway [172].

Specific contribution of miRNAs to LV cardiomyopathy induced by AS was further confirmed by Ikeda et al. [173, 174]. They reported 87 differentially expressed miRNAs (among 428 examined) in LV samples of AS patients compared to samples from diseased hearts with DCM and ischemic cardiomyopathy/ICM and the non-failing control group [173, 174]. Among the miRNAs with known cardiac-enriched expression (miR-1, miR-133, and miR-208), miR-1 was downregulated in DCM and AS and tended to be downregulated in ICM patients while the expression of miR-133 and miR-208 were not significantly changed [173, 174]. In addition, the miR-214 exhibited the up-regulation in all three disease groups while the miR-19a and miR-19b were the most down-regulated miRs in DCM and AS, but not in ICM patients [173, 174]. MiR-24 has also been identified as significantly upregulated in AS patient group [173, 174]. Importantly, differential expression of 13 of these miRNAs was specific to AS, while eight miRNAs exhibited differential expression in cardiomyopathy groups (ICM and DCM) that did not overlap with the expression of AS specific miRs thus suggesting that altered expression of these miRNAs reflects distinct disease mechanisms or disease stage in AS compared with cardiomyopathy samples [173, 174].

Another miRNA-wide microarray study reported by Gallego et al. detected differential expression of 70 miRNAs (64 downregulated and 6 upregulated) between control subjects and two clusters of AS patient identified according to cardiomyocyte apoptotic index [175]. Among them miR-10b, miR-125b-2* and miR-338-3p were inversely correlated with cardiomyocyte apoptotic index [175]. They concluded that myocardial downregulation of miR-10b may be involved in increased cardiomyocyte apoptosis in AS patients, probably through the upregulation of apoptotic peptidase activating factor 1 (*APAF1*) gene expression, thus contributing to cardiomyocyte damage and to the development of heart failure [175].

Finally, Beaumont et al. reported reduced expression of miR-133 and miR-19b in the myocardial and serum samples of AS patients when compared to controls [176]. Moreover, both myocardial and serum miR-19b levels were found inversely correlated with expression levels of lysyl oxidase (LOX), collagen cross-linking and left ventricular stiffness in AS patients, particularly in patients with heart failure [176]. In addition, the expression levels of miR-29b, miR-1, miR-208a and miR-499-5p was under the limit while no differences were found for miR-21 levels between serum samples from AS patients and control subjects [176].

Taken together the above data provide clear evidence that aberrantly expressed miRNAs are implicated in a diverse spectrum of pathophysiological pathways leading to AS development and progression, and consequently to pressure overload-induced myocardial remodeling and hypertrophy. This is obviously just a beginning of story with the list of novel miRNA players and the previously unknown roles for the already existent ones growing at a steady pace.

## Conclusion

Herein we presented a growing body of experimental evidences to support the key role of epigenetic alterations in the etiology and progression of AS. Evidently they participate in crucial disease-prone phenotype changes of aortic valve cells, and regulate key processes underlying AS-induced valvular tissue remodeling and maladaptive myocardial hypertrophy, i.e., fibrosis, calcification, LV remodeling, and inflammation. As can be seen, alteration of DNA methylation marks may contribute to production of proinflammatory mediators (ALOX5 induced LTB4 production), and have role in osteogenic transformation of VICs (H19 induced NOTCH1 downregulation and subsequent upregulation of RUNX2, BMP2, and OCN) while some of them demonstrate promising biomarker potentials for the prediction of AS status (analysis of dried blood spots of neonatal AS patients). Proinflammatory and osteogenic role was also demonstrated for histon code marks (mediated by alterations of SIRTs gene expression), and their involvement in AS-induced pathological remodeling of left ventricular myocardium was also clearly established (e.g., reexpression of the fetal gene programs induced by altered expression of *JARID2* and *DPF3* genes). Apparently most studies were focused on the role of small non-coding miRNAs demonstrating their essential role in some of the key processes responsible for disease progression such as the phenotypic alterations of valvular endothelial and interstitial cells under pathologically altered blood born mechanical forces, induction of proinflammatory pathways, and osteogenic transdifferentiation of VICs (Table 4). Moreover, they seem to be crucial for the disease-prone role of monocyte-macrophage and COPC cells and have established role in regulation of myocardial fibrosis and LVH. Most importantly, specific miRNA signatures (e.g., plasma levels of miR-21, miR-210, and miR-378), in combination with clinical and functional imaging parameters, could represent useful non-invasive biomarkers of disease progression or recovery after aortic valve replacement. Less is known about the ATP-dependent chromatin remodeling processes and long noncoding RNAs and their extent and potential role in subclinical and clinical manifestation of AS have yet to be examined and experimentally validated in both small and large scale human and animal studies. Also, we currently see only the very tip of histone and DNA methylation marks lying under the surface of AS patohistogenesis. Since all epigenetic mechanisam in a given cell and tissue structure are mutually interdependent as clearly represented by Theodoris et al. A better understanding of epigenome landscape in native and infiltrating aortic valve cells and affected myocardium will certainly shed new insights into all aspects of AS pathology and add important incremental diagnostic and prognostic informations useful for risk stratification and patient management. Even more, since all known epigenetic marks are potentially reversible this perspective is especially exciting given the potential for development of successful and non-invasive therapeutic intervention and reprogramming of cells at the epigenetic level even in the early stages of disease progression.

## Abbreviations

ACTA2/ αSMA: alpha-smooth muscle actin
AFAP1: Actin filament associated protein 1
AFAP1-AS1: AFAP1 Antisense RNA 1
ALOX5/5LO: 5-lipoxygenase
ALOXAP/FLAP: 5-LO activating protein
ALP: Alkaline phosphatase
ANF: Atrial natriuretic factor
ANGII/AGT: Angiotensin 2
APAF1: Apoptotic peptidase activating factor 1
APOA1: Apolipoprotein A1
APOA5: Apolipoprotein A5
APO-AS1: APOA1-antisense transcript 1
APOB: Apolipoprotein B
APOE: Apolipoprotein E
AS: Aortic valve stenosis
ASc: Aortic valve sclerosis
AVECs: Aortic valve endothelial cells
aVICs: Activated aortic valve interstitial cells
AVICs: Aortic valve interstitial cells
AVR: Aortic valve replacement
BAF: BRG/BRM-associated factor
BAV: Bicuspid aortic valve
BGLAP/OCN: Bone gamma-carboxyglutamate (Gla) protein; osteocalcin
BMP: Bone morphogenetic protein
BMPR2: Bone morphogenic protein receptor type 2
BRG1/SMARCA4: Brahma-related gene 1; SWI/SNF related, matrix associated, actin dependent regulator of chromatin, subfamily a, member 4
BRM/SMARCA2: Brahma; SWI/SNF related, matrix associated, actin dependent regulator of chromatin, subfamily a, member 2
CAD: Coronary artery disease
CARMEN: Cardiac mesoderm enhancer-associated noncoding RNA
CCL: Chemokine (C-C motif) ligand
CH: Cardiac hypertrophy
CHAST: Cardiac hypertrophy-associated lncRNA transcript
CMAI: Cardiomyocyte apoptotic index
COPCs: Circulatory osteoprogenitor cells
CSNK2: Casein kinase 2
CTGF: Connective tissue growth factor
CVD: Cardiovascular diseases
DCM: Dilated cardiomyopathy
DICER1: Dicer 1, Ribonuclease III
DLX5: Distal-less homeobox 5
DNMT: DNA (Cytosine-5-)-methyltransferase
DPF3/BAF45C: Double PHD fingers 3/BRG1-associated factor 45C
DUSP27: Dual-specificity phosphatase 27
ECM: Extracellular matrix
ECs: Endothelial cells
EDN1: Endothelin 1
EGFR: Epidermal growth factor receptor
ENG: Endoglin
ETS1: ETS proto-oncogene 1
EZH: Enhancer of zeste
FABP: Fatty acid-binding protein
FOXO1: Forkhead box O1
GATA4: Growth arrest and DNA damage-inducible protein 45
H3K27ac: Acetylation of histone H3 at lysine 27
H3K27me2/3: Methylation of histone H3 lysine 27
H3K27me3: Trimethylation of histone H3 at lysine 27
H3K4me1: Monomethylation of histone H3 at lysine 4
H3K4me3: Trimethylation of histone H3 at lysine 4
HAT: Histone acetyltransferase
HDAC: Histone deacetylase
HEY1/2: Hes related family BHLH transcription factor with YRPW motif 1/2
HF: Heart failure
hiPSC: Human induced pluripotent stem cell
HNCM: Hypertrophic non-obstructive cardiomyopathy
HOCM: Hypertrophic obstructive cardiomyopathy
HOTAIR: HOX transcript antisense RNA
ICM: Ischemic cardiomyopathy
IGF: Insulin-like growth factor
JARID2: Jumonji and AT-rich interaction domain containing 2
LA: LDLR^-/-^APOB^100/100^ mice
LDLR: Low density lipoprotein
lncRNAs: Long non-coding RNAs
LOX: Lysyl oxidase
LTA4H: Leukotriene A4 hydrolase
LTB4: Leukotriene B4
LTC4S: Leukotriene C4 synthase
LV: Left ventricle
LVH: Left ventricular hypertrophy
MALAT1: Metastasis-associated lung adenocarcinoma transcript 1
MAP3K7/TAK1: Mitogen-activated protein kinase kinase kinase 7
MF: Myocardial fibrosis
MGP/MGLAP: Matrix γ-carboxyglutamate (Gla) protein
microRNAs/miRNAs/miRs: Small non-coding RNAs
MMP: Matrix metalloproteinase
MSCs: Mesenchymal stem cells
MSX2: MSH homeobox 2
MYH2: Myosin heavy chain 2
MYL7: Myosin light chain 7
ncRNAs: Non-coding RNAs
NFYC: Nuclear transcription factor gamma subunit c
NOTCH1: Notch/drosophila/homolog 1/translocation-associated
NPPA/ANP: Natriuretic peptide A
NT-proBNP: N-terminal probrain natriuremic peptide
obVICs: Osteoblast-like aortic valve interstitial cells
PARP: Poly (ADP ribose) polymerase
PCSK9: Proprotein convertase and subtilisin/knexin-type 9
PDCD4: Programmed cell death 4 (neoplastic transformation inhibitor)
PF: Pericardial fluid
PO: Pressure overload
POSTN: Periostin
PPARγ: Peroxisome proliferator-activated receptor γ
PRC2: Polycomb repressive complex 2
PRINS: Psoriasis associated non-protein coding RNA induced by stress
PTEN: Phosphatase and tensin homologue
PTENP1: Phosphatase and tensin homolog pseudogene 1
PTENP1-AS: PTENP1-antisense RNA
qVICs: Quiescent aortic valve interstitial cells
RECK: Reversion inducing cysteine rich protein with kazal motifs
RETN: Resistin
ROR: Regulator of reprogramming, also named lncRNA
ROS: Reactive oxygen species
RUNX: Runt-related transcription factor 1
SAHA: Suberoylanilide hydroxamic acid
SIRT: Silent information regulator-two
SM22α: Smooth muscle protein 22-alpha
SMAD: SMAD family member
SMCs: Smooth muscle cells
SOD: Superoxide dismutase
SOST: Sclerostin
SOX2: SRY-Box 2
SOX2-OT: SOX2 overlapping transcript
SP7/OSX: Osterix
SPP1/OPN: Osteopontin, secreted phosphoprotein 1; bone sialoprotein 1
SWI/SNF: SWItch/sucrose non-fermentable)-like ATP-dependent BAF chromatin remodeling complex chromatin remodeling complex
TAC: Transverse aortic constriction
TAV: Tricuspid aortic valve
TAVR: Transcatheter aortic valve replacement
TBX3: T-box 3
TGFBR1: Transforming growth factor beta receptor 1
TGFβ: Transforming growth factor beta
TIMP3: Tissue inhibitor of metalloproteinases
TWIST1: Twist-related protein 1
TXNRD2: Thioredoxin reductase 2
VICs: Aortic valve interstitial cells
VSMCs: Vascular smooth muscle cells
WISP2: WNT1 inducible signaling pathway protein 2
WISPER: WISP2 super-enhancer associated RNA
WT: Wild type
